# Stress-Doped Interface Synergy: Unraveling the Atomic-Scale Corrosion Initiation of Al/Al_2_Cu Interfaces with Fe–Si Additions in Chloride Environments

**DOI:** 10.3390/ma19051026

**Published:** 2026-03-06

**Authors:** Shuang Li, Wenyan Wang, Jingpei Xie, Aiqin Wang, Zhiping Mao, Wendong Qin, Qingyuan Guo

**Affiliations:** 1 School of Materials Science and Engineering, Henan University of Science and Technology, Luoyang 471023, China; 2 Digital Molding Engineering Research Center of Tungsten and Molybdenum Materials in Henan Province, Luoyang 471822, China; 3 State Key Laboratory of Light Superalloys, Luoyang 471023, China

**Keywords:** interface, adsorption, galvanic corrosion, electron work function, differential charge density, first-principles calculation

## Abstract

In this study, first-principles calculations were employed to systematically investigate the adsorption of Cl^−^ on Al_2_Cu(110) surfaces, clean Al(111)/Al_2_Cu(110) interfaces, and Fe/Si-doped interfaces, as well as the influence of strain on interfacial electronic structure and corrosion activity. When Cl^−^ is adsorbed on Al sites, the bonding between Cl and Al exhibits strong ionic characteristics with localized charge transfer, while adsorption on Cu sites is characterized by more delocalized, covalent interactions. This competition dictates the site-dependent stability of adsorption. Through geometric–electronic synergy, the interface functions as both a “Cl^−^ enrichment zone” and an “activity source,” significantly favoring Cl^−^ adsorption at high-activity anodic sites such as Al-hole and Al-bridge. Conversely, Cu-top sites maintain a high work function and an inert cathodic nature, facilitating the formation of efficient micro-galvanic couples across the interface. Moreover, Fe/Si doping further modulates the interfacial electronic landscape: Si serves as an effective strengthening element due to its low substitution energy and high stability, while Fe primarily forms a solid solution on the Al side, potentially introducing galvanic corrosion risks. Stress analysis indicates that tensile strain systematically enhances surface activity by lowering the work function, while compressive strain non-monotonically influences corrosion through a three-stage mechanism involving the “densification–cracking–plastic relaxation” of the passive film. These findings elucidate the atomistic origins of corrosion initiation at Cu–Al composite interfaces and provide a theoretical foundation for enhancing corrosion resistance through alloy design and strain engineering.

## 1. Introduction

Copper–aluminum (Cu–Al)-laminated composites are indispensable materials for modern power electronics, electric vehicle battery systems, and aerospace components due to their high electrical and thermal conductivity and low density, providing critical functional advantages [1,2,3,4]. During service, these materials are constantly subjected to atmospheric conditions. Corrosive species in the atmosphere include ions such as chloride (Cl^−^), sulfate (SO_4_^2−^), nitrate (NO_3_^−^), hydrogen (H^+^), and hydroxide (OH^−^), which cause the corrosive deterioration of the materials. Consequently, interfacial corrosion plays a crucial role in restricting the long-term service reliability of these composites [5,6]. The performance of these composites is fundamentally dictated by the stability of their internal interfaces [7,8,9,10]. During manufacturing or when exposed to elevated temperatures, brittle intermetallic compounds (IMCs), mainly Al_2_Cu, AlCu, and Al_4_Cu_9_, are generated at the Al/Cu interface [11,12,13,14]. Among these phases, the θ-phase Al_2_Cu typically nucleates first and frequently dominates the interfacial microstructure, exerting a decisive influence on both mechanical integrity and corrosion behavior [15,16].

Intrinsic electrochemical potential differences among Al, Cu, and their IMCs generate local galvanic couples, rendering the interface exceedingly susceptible to localized corrosion in aggressive environments [17,18,19,20]. Numerous experimental observations confirm that IMC particles in Al alloys, particularly Al_2_Cu, act as preferred cathodic sites and accelerate the nucleation and expansion of pits in the adjacent anodic Al matrix [21,22,23,24]. Although these macroscopic corrosion phenomena are well established, the atomic-scale mechanisms governing the incipient corrosion stage, and specifically the interactions between Cl^−^ and the complex Al/Al_2_Cu heterointerface, remain poorly understood. This critical knowledge gap hinders the rational design of efficient corrosion-resistant interfaces.

Recent advances underscore the importance of fundamental electronic descriptors for unraveling corrosion mechanisms. The electron work function, a metric reflecting the ease of electron emission from a surface, correlates strongly with both corrosion potential and susceptibility to localized corrosion [25,26]. First-principles calculations have emerged as a premier tool for retrieving these descriptors at the atomic scale, enabling the precise prediction of the work functions, adsorption energies, and interfacial charge redistribution [27,28]. This approach has been successfully applied to investigate the stability of IMCs [29,30] and to compute Volta potential differences as proxies for galvanic activity [31,32], demonstrating its capacity to elucidate the fundamental mechanisms governing corrosion.

Nevertheless, a comprehensive atomic-scale exploration of chloride interactions at the Al/Al_2_Cu heterointerface remains incomplete, despite its critical role in revealing corrosion initiation. Critically, there is still a lack of quantitative mechanistic understanding regarding two pervasive and unavoidable factors: common alloying elements such as Fe and Si that are extensively utilized to enhance strength and processability of 8011 aluminum alloys [33], and the mechanical strain that is inherent to material fabrication and service. The lack of understanding of how these factors influence the modulation of interfacial electronic structure, chloride adsorption, and incipient corrosion is a significant barrier to the strategic design of corrosion-resistant Cu–Al composites.

To address these challenges, we carried out a systematic review of relevant literature through a structured and reproducible search strategy across major academic databases including Web of Science and Scopus. The core search terms included “Al/Cu interface,” “intermetallic corrosion,” “chloride adsorption,” and “first-principles calculations.” Our analysis reveals that although previous studies have characterized galvanic effects and interfacial phase formation, there is a lack of dedicated atomic-scale investigations that explicitly link interfacial electronic structure, elemental doping, mechanical strain, and chloride adsorption.

In this study, density functional theory (DFT) was employed to establish a predictive atomic-scale model for corrosion initiation at the Al/Al_2_Cu heterointerface. Building upon conventional adsorption-oriented investigations, this study systematically incorporates three decisive factors that govern interfacial corrosion behavior. We elucidate how the buried heterointerface modulates the adsorption of Cl^−^ on the neighboring Al_2_Cu surface through interfacial electronic effects, quantify the impacts of Fe or Si substitution on local bonding characteristics and electrochemical activity, and evaluate the regulation of corrosion driving forces by mechanical strain through lattice and electronic structure reconfiguration. By combining adsorption energetics, work function, differential charge density, and density of states (DOS) analyses, the synergistic contributions of geometric and electronic features to Cl^−^ adsorption stability and the redistribution of surface active sites were unraveled. This integrated computational approach not only clarifies the intrinsic mechanisms by which Fe/Si doping and mechanical strain control interfacial corrosion susceptibility, but also offers a theoretical framework for the rational and strategic design of corrosion-resistant heterointerfaces through targeted alloying and strain engineering.

## 2. Models and Methods

### 2.1. Computational Models

#### 2.1.1. Surface Adsorption Model

The geometric optimization of bulk Al_2_Cu was performed, and a low-index (110) surface model was constructed by using Materials Studio 2019 (64-bit). The selection of the Cu-terminated Al_2_Cu(110) surface was based on our previous systematic first-principles calculations. In our earlier research, we evaluated four distinct termination models of the Al_2_Cu/Al interface and found that the Cu-terminated slab in contact with Al(111) was the most thermodynamically stable configuration, with the lowest interfacial energy (−0.01 to 0.41 J·m^−2^) and the highest work of adhesion (2.76 J·m^−2^) [34]. Additional electronic structure analysis revealed that the stability was attributed to strong charge accumulation at the interface, resonant orbital hybridization between Cu and Al, and a combination of metallic and covalent bonding responsible for reinforcing interfacial cohesion.

**Figure 1:**
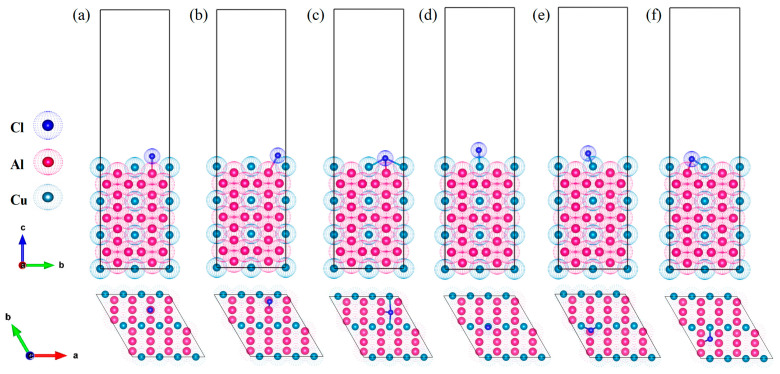
Atomic models of Cl^−^ adsorption on the Al_2_Cu(110) surface: (**a**) Al-top site, (**b**) Al-bridge site, (**c**) Al-hole site, (**d**) Cu-top site, (**e**) Cu-bridge site, and (**f**) Cu-hole site.

Furthermore, in this study, Cl^−^ adsorption behavior and its impact on surface properties were systematically investigated by considering six characteristic adsorption sites on the Al_2_Cu(110) surface [35]. These sites were categorized based on the type of surface metal atom (Al or Cu) and the local coordination geometry. Three sites are associated with surface Al atoms: the Al-top site, where Cl^−^ gets adsorbed directly above a single surface Al atom; the Al-bridge site, where Cl^−^ binds at the midpoint between two adjacent surface atoms including one Al; and the Al-hollow site, a multi-coordinated site where Cl^−^ occupies a surface cavity surrounded by multiple atoms, at least one of which is Al. The three remaining sites are related to surface Cu atoms: the Cu-top site, where Cl^−^ is adsorbed directly above a single Cu atom; the Cu-bridge site, where Cl^−^ is located between two adjacent surface atoms, one of which is Cu; and the Cu-hollow site, a multi-coordinated cavity site surrounded by multiple atoms, at least one of which is Cu. Corresponding adsorption models were constructed to represent all dominant Cl^−^ adsorption configurations on this surface. The surface was modeled using a periodic slab approach, as illustrated in Figure 1. The bottom three atomic layers were kept fixed to represent the bulk substrate, while the top four layers and adsorbed Cl^−^ were completely relaxed to reproduce realistic surface conditions. A 15 Å vacuum layer was included above the slab to prevent any artificial interactions between periodic images. All computational parameters were validated via systematic convergence tests to verify the reliability and accuracy of the results.

Calculations were performed by using the Vienna Ab initio Simulation Package (VASP 5.4.4), a quantum mechanical software package based on DFT. Periodic boundary conditions were applied, and the electron–ion interactions and electron exchange–correlation energy were described by using the projector-augmented wave pseudopotential. Valence electrons were explicitly considered as follows: Al (3s^2^3p^1^), Cu (3d^10^4s^1^), and Cl (3s^2^3p^5^). The Perdew–Burke–Ernzerhof generalized gradient approximation was employed for exchange-correlation functional calculations, and the calculation accuracy was set to “Medium” (Med). Calculations were performed with a plane-wave energy cutoff of 500 eV, energy convergence criteria of 1 × 10^−5^ eV, and force convergence criteria of 0.02 eV·Å^−1^. For geometric optimization and electronic structure calculations, Brillouin zone sampling was carried out using κ vectors of 4 × 4 × 1 and 6 × 6 × 1, respectively.

#### 2.1.2. Interface Model

The Al(111) and Al_2_Cu(110) surfaces were selected based on their thermodynamic stability and experimental prevalence. In face-centered cubic Al, the (111) plane is the most densely packed and exhibits the lowest surface energy, making it the most thermodynamically stable facet. This is corroborated by experimental evidence [36]. The X-ray diffraction (XRD) analysis reveals that the Al(111) diffraction peak shows the highest intensity, and transmission electron microscopy (TEM) result confirms it as the dominant exposed facet. Both XRD and TEM analyses confirm that the (110) plane is the dominant surface in Al_2_Cu. Previous research has shown that this plane is the most readily activated slip system under tensile loading, with a Schmid factor of 0.4714, highlighting its importance as a highly active interface under service conditions.

**Figure 2:**
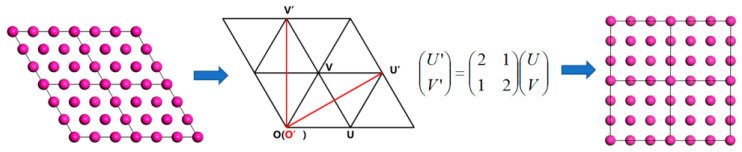
Schematic illustration of the √n × √n surface cutting method for Al(111).

In Materials Studio 2019, the Al(111) surface was cleaved along specific crystallographic directions using the “√n × √n” method to create a stable, coherent interface with minimal lattice mismatch, as illustrated in Figure 2. Slab thicknesses were determined through systematic convergence tests as outlined in our previous research. A 7-layer model was utilized for Al(111), focusing on atomic relaxation primarily in the top three layers, while the central layers exhibited a bulk-like structure in slabs with seven or more layers. A 13-layer symmetric slab was employed for the polar Al_2_Cu(110) surface. This thickness was selected because interlayer spacing converged after 13 layers, ensuring that the slab accurately reproduced the bulk electronic structure [37].

**Figure 3:**
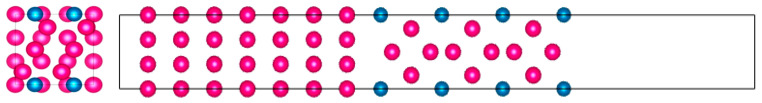
Atomic model of the Al(111)/Al_2_Cu(110) interface.

The optimized 7-layer Al(111) supercell was aligned with the 13-layer, Cu-terminated Al_2_Cu(110) slab, resulting in minimal lattice mismatches of 1.7% and 0.3% along the X and Y directions, respectively. These values satisfy the criteria for a coherent interface, allowing for the construction of an Al(111)/Al_2_Cu(110) interface model depicted in Figure 3. A 20 Å vacuum layer was included above the slab to avoid any artificial interactions between periodic images, and its adequacy was verified through convergence tests. To comprehensively characterize Cl^−^ adsorption, six distinct adsorption sites on the Al_2_Cu(110) surface were investigated, and categorized by identifying the surface metal atom (Al or Cu) and the local coordination geometry.

### 2.2. Research Principles and Methods

#### 2.2.1. Surface Energy Calculation Method

As a key parameter for evaluating surface stability, surface energy quantifies the energy difference between a surface and its bulk phase per unit area. In general, a lower surface energy indicates higher surface stability, indicating that a material is less susceptible to corrosion. The surface energy of Cu–Al composites can be theoretically estimated as follows [38]:

(1) $$E_{surf}=\frac{1}{2A}(E_{slab}-N_{Al}E_{\mathrm{Al}}^{slab}-N_{Cu}E_{\mathrm{Cu}}^{slab}-PV-TS)$$

where E*_slab_* represents the total energy of various low-index crystal plane models of relaxed Cu–Al IMCs. $E_{\mathrm{Al}}^{slab}$ and $E_{Cu}^{slab}$, respectively, denote the chemical potentials of Al and Cu atoms in the surface models of these compounds. N_Al_ and N_Cu_ represent the quantities of Al and Cu atoms present in the surface models, separately. *A*, *P*, *V*, *T*, and *S* represent the surface area, pressure, volume, temperature, and entropy of the surface model, respectively. At absolute zero or under low-temperature conditions, the *PV* and *TS* terms can be disregarded. In this study, the chemical potentials of atoms in the surface models of the IMCs are assumed to be equivalent to those in the bulk structures. For example, the bulk chemical potential of Al_2_Cu can be expressed as follows:

(2) $$E_{{\mathrm{Al}}_{2}\mathrm{Cu}}^{bulk}\;=\;2E_{\mathrm{Al}}^{slab}\;+\;E_{\mathrm{Cu}}^{slab}$$

Using Formulas (1) and (2), the surface energy of Al_2_Cu can be described as follows:

(3) $$E_{surf}({\mathrm{Al}}_{2}\mathrm{Cu})=\frac{1}{2A}\left[E_{slab}-N_{Cu}E_{{\mathrm{Al}}_{2}\mathrm{Cu}}^{bulk}-(N_{Al}-2N_{Cu})E_{\mathrm{Al}}^{slab}\right]$$

For the non-stoichiometric polar Al_2_Cu surface (where 2N_Cu_ ≠ N_Al_), the chemical potentials of Al and Cu must be considered when calculating its surface energy. In this context, determining the range of variation in the elemental chemical potentials is essential for investigating the surface energy of polar surfaces, which can be calculated as follows:

The chemical potentials of Al and Cu are defined as follows:

(4) $$∆E_{Al}=E_{\mathrm{Al}}^{slab}-E_{\mathrm{Al}}^{bulk};\;∆E_{Cu}=E_{Cu}^{slab}-E_{Cu}^{bulk}$$

where $E_{\mathrm{Al}}^{bulk}$ and $E_{Cu}^{bulk}$ denote the total energies of the bulk Al and Cu phases, respectively.

Substituting Formula (4) into Formula (3) yields the following equation:

(5) $$E_{surf}({\mathrm{Al}}_{2}\mathrm{Cu})=\frac{1}{2A}\left[E_{slab}-N_{Cu}E_{{\mathrm{Al}}_{2}\mathrm{Cu}}^{bulk}-(N_{Al}-2N_{Cu})∆E_{Al}-(N_{Al}-2N_{Cu})E_{\mathrm{Al}}^{bulk}\right]$$

where $E_{{\mathrm{Al}}_{2}\mathrm{Cu}}^{bulk}$ represents the total energies of the Al_2_Cu bulk materials.

Equation (6) shows that the chemical potentials of Al and Cu atoms in the surface models must be lower than those in the bulk for preventing the surface model from becoming unstable and decomposing into elemental substances.

(6) $$∆E_{Al}=E_{\mathrm{Al}}^{slab}-E_{\mathrm{Al}}^{bulk}\leq 0;\;∆E_{Cu}=E_{Cu}^{slab}-E_{Cu}^{bulk}\leq 0$$

Furthermore, the formation enthalpy of Al_2_Cu can be expressed as follows:

(7) $$E_{{\mathrm{Al}}_{2}\mathrm{Cu}}^{bulk}=2E_{\mathrm{Al}}^{bulk}+E_{\mathrm{Cu}}^{bulk}+∆H_{f}^{0}\left({\mathrm{Al}}_{2}\mathrm{Cu}\right)$$

Combining Formulas (2) and Formulas (7) yields:

(8) $$2E_{\mathrm{Al}}^{slab}+E_{\mathrm{Cu}}^{slab}=2E_{\mathrm{Al}}^{bulk}+E_{\mathrm{Cu}}^{bulk}+∆H_{f}^{0}\left({\mathrm{Al}}_{2}\mathrm{Cu}\right)$$

Comparison of Equation (4) and Formula (8) yields:

(9) $$∆H_{f}^{0}({\mathrm{Al}}_{2}\mathrm{Cu})=2∆E_{Al}+∆E_{Cu}$$

Equations (4) and (6) can be substituted into Equation (9) to obtain the following Equation (10):

(10) $$∆H_{f}^{0}({\mathrm{Al}}_{2}\mathrm{Cu})=2∆E_{Al}+∆E_{Cu}\leq 0$$

In this study, the calculated value of $∆H_{f}^{0}({\mathrm{Al}}_{2}\mathrm{Cu})$ is −0.578 eV, showing a slight deviation from a previously reported theoretical value of −0.553 eV [39] and an experimental value of −0.546 eV [40]. By combining Equations (9) and (10), the range of chemical potentials of Al and Cu atoms in the Al_2_Cu surface model can be derived as follows:

(11) $$-0.578\leq 2∆E_{Al}+∆E_{Cu}\leq 0$$

When $∆E_{Al}=0$, the surface energy of Al_2_Cu varies linearly with ∆E_Cu_; conversely, for $∆E_{Cu}=0$, it varies linearly with ΔE_Al_. Specifically, when $∆E_{Al}=0$, the surface energy of Al_2_Cu is 0.536 J·m^−2^; and for $∆E_{Al}=-0.289$ eV, it decreases to 0.092 J·m^−2^. Notably, the surface energy is strongly influenced by the bulk energy of Al_2_Cu.

#### 2.2.2. Substitution Energy Calculation Method

Substitution energy (ΔE_sub_) is the energy change required to exchange one atom for another at a specific interface position. A positive ΔE_sub_ value indicates that energy is required for the substitution, while a negative value suggests that energy is released, indicating a favorable substitution process. Substitution energies in this study were calculated as follows [41]:

(12) $$∆E_{\mathrm{sub}}=(E_{i\to j}^{Al/Al_{2}Cu}-E^{Al/Al_{2}Cu})-(\mu_{i}-\mu_{j})$$

where i is the dopant element Fe or Si and j is the Al or Cu element in the Al/Al_2_Cu phase interface structure. $E_{i\to j}^{Al/{\mathrm{Al}}_{2}Cu}$ denotes the total energy of the Al/Al_2_Cu phase interface after the substitution of *j* atom by *i* dopant atom. $E^{Al/{\mathrm{Al}}_{2}Cu}$ represents the total energy of the Al/Al_2_Cu phase interface in the undoped state. *μ_i_* and *μ_j_* represent the chemical potentials of atoms *i* and *j*, respectively. The chemical potential of a pure substance is equal to its molar Gibbs free energy. The Gibbs free energy of Al_107_ can be found in the Langevin Handbook of Chemistry [42]. According to the literature [41], the calculation of this chemical potential can be approximated by using a 3 × 3 × 3 unit cell to determine the energy difference between Al_107_ and pure Al.

(13) $$\mu (i)=E(Al_{107}i)-107\mu (Al)$$

$\mu_{Si}=-5.437$; $\mu_{Al}=-3.742$; $\mu_{Fe}=-7.696$; $\mu_{Cu}=-3.754$.

#### 2.2.3. Adhesion Work Calculation Method

Adhesion work (*W*_ads_) is defined as the reversible work required per unit area to separate a two-phase interface into two free surfaces. Thus, *W*_ads_ serves as an indicator of interfacial bonding strength, with a higher *W*_ads_ value signifying stronger atomic bonding at the interface. The value of *W*_ads_ can be calculated as follows [43]:

(14) $$W_{\mathrm{ads}}=\frac{E_{slab}^{Al(111)}+E_{slab}^{Al_{2}Cu(110)}-E_{\mathrm{int}}^{Al(111)/Al_{2}Cu(110)}}{A}$$

where $E_{slab}^{Al(111)}$ represents the total energy of the Al(111) surface slab in the Al(111)/Al_2_Cu(110) interface model, $E_{slab}^{{\mathrm{Al}}_{2}Cu(110)}$ represents the total energy of the Al_2_Cu(110) surface slab in the Al(111)/Al_2_Cu(110) interface model, $E_{int}^{Al(111)/{\mathrm{Al}}_{2}Cu(110)}$ denotes the total energy of the Al(111)/Al_2_Cu(110) interface model, and A is the area of the interface.

#### 2.2.4. Interfacial Energy Calculation Method

Interfacial energy is a crucial parameter for determining the stability of interfaces. When two distinct solid materials are used to form an interface, the interfacial energy is typically positive due to structural mismatch at the interface, which can induce mismatch strain. Lower positive interfacial energy values are indicative of greater stability. Noteworthy, interfaces exhibiting negative interfacial energies are thermodynamically unstable. A negative interfacial energy can act as a driving force for atomic diffusion near the interface, facilitating interfacial alloying. In this study, the interfacial energy of Al(111)/Al_2_Cu(110) was calculated as follows [44]:

(15) $$\gamma_{\mathrm{int}}=\sigma_{Al(111)}+\sigma_{Al_{2}Cu(110)}-W_{\mathrm{ads}}$$

where $\sigma_{Al(111)}$ and $\sigma_{{Al}_{2}Cu(110)}$, respectively, denote the surface energies of the Al(111) and Al_2_Cu(110) free surfaces, while $\sigma_{{Al}_{2}Cu(110)}$ is not a fixed value but varies within a range of 0.092 to 0.536 J·m^−2^. *W*_ads_ represents the interfacial adhesion energy formed by free surface bonding.

#### 2.2.5. Adsorption Energy Calculation Method

Adsorption energy (*E*_ads_) is a crucial physical parameter that quantifies the strength of interactions among gas molecules, atoms, ions, and solid surfaces [45]. *E*_ads_ was calculated within a first-principles framework integrated with an implicit solvation model. It is defined as the energy difference between the adsorption system and the sum of the energy of the clean surface and the reference chemical potential of a chlorine atom (*E*_Cl_) in the aqueous electrochemical environment, as expressed by the following equation:

(16) $$E_{\mathrm{ads}}=E_{\mathrm{total}}-(E_{\mathrm{surface}}+E_{\mathrm{Cl}})$$

In this equation, *E*_total_ and *E*_surface_ represent the total energies of the Cl^−^ adsorbed system and the pristine surface, respectively, obtained from single-point calculations using the VASPsol implicit solvation model (with the dielectric constant of water, ε = 78.4) to approximate the aqueous electrochemical environment. The pivotal term, $E_{\mathrm{Cl}}$, represents the reference chemical potential of a chlorine adsorbate. It corresponds to the chemical potential of a chlorine atom in its standard state within the electrolyte, effectively defining the energy baseline for a Cl^−^ ion in solution at the potential of the standard hydrogen electrode (SHE, U = 0 V).

Determining $E_{\mathrm{Cl}}$ in a physically meaningful manner requires coupling DFT energies to standard experimental electrochemical data via a thermodynamic cycle, a well-established approach in computational electrocatalysis [46]. The construction is as follows:

(17) $$E_{Cl}=\frac{1}{2}E{(Cl_{2})}_{\mathrm{gas}}+\frac{1}{2}∆G_{\mathrm{solv}}(Cl_{2})+\mathrm{EA}(\mathrm{Cl})+\frac{1}{2}E(H_{2}{)}_{\mathrm{gas}}-∆G_{H^{+}/\frac{1}{2}H_{2}}^{\circ }$$

Here, the constituent terms and their sources are:

$E{(Cl_{2})}_{\mathrm{gas}}$ and $E(H_{2}{)}_{\mathrm{gas}}$, the DFT-calculated total energies of an isolated Cl_2_ molecule and an H_2_ molecule in the gas phase, respectively, serving as calculable references.

EA(Cl), the experimental electron affinity of a chlorine atom, taken as 3.613 eV [47].

$∆G_{H^{+}/\frac{1}{2}H_{2}}^{\circ }$, the absolute potential of the standard hydrogen electrode, adopted as the standard value of 4.440 eV [48]. This key constant aligns the DFT energy scale (referenced to vacuum) with the experimental electrochemical scale (referenced to SHE).

$∆G_{\mathrm{solv}}(Cl_{2})$, the standard solvation free energy of Cl_2_, which is thermodynamically equivalent to the standard Gibbs free energy of formation of dissolved chlorine, ${∆}_{f}G^{\circ }[Cl_{2}(aq)]$. This value is derived from standard electrochemical relations:

(18) $$∆G_{\mathrm{solv}}(Cl_{2})={∆}_{f}G^{\circ }[Cl_{2}(aq)]=2\times {∆}_{f}G^{\circ }[Cl^{-}(aq)]-(-nFE^{\circ })$$

where ${∆}_{f}G^{\circ }[Cl^{-}(aq)]$ = −131.2 kJ/mol is the standard Gibbs free energy of formation of the aqueous chloride ion [49]; $E^{\circ }$ =1.358 V is the standard electrode potential for the half-reaction $Cl_{2}(aq)+2E^{-}⇌2Cl^{-}(aq)$; n = 2; and F is the Faraday constant.

The value calculated from Equation (18) corresponds to the standard molar Gibbs free energy of formation $∆G_{\mathrm{solv}}(Cl_{2})$ defined for an ideal 1 mol/L solution. In electrochemical conventions, however, the standard potential ($E^{\circ }$) of the Cl_2_/Cl^−^ redox couple is calibrated against a distinct reference state. This state corresponds to $Cl_{2}(aq)$ in Henry’s law equilibrium with 1 bar Cl_2_(g), extrapolated to a hypothetical 1 mol/L concentration. For consistency with established electrochemical frameworks and tabulated standard thermodynamic data, this work adopts the $∆G_{\mathrm{solv}}(Cl_{2})$ value, corresponding to this gas-equilibrated reference state, for all calculations. The final $E_{\mathrm{Cl}}$ is obtained by inserting this result along with the other constants and the calculated $E{(Cl_{2})}_{\mathrm{gas}}$ and $E(H_{2}{)}_{\mathrm{gas}}$ into Equation (17).

## 3. Results and Discussion

### 3.1. Adsorption Properties of Cl^−^ at the Al_2_Cu(110) Surface

**Figure 4:**
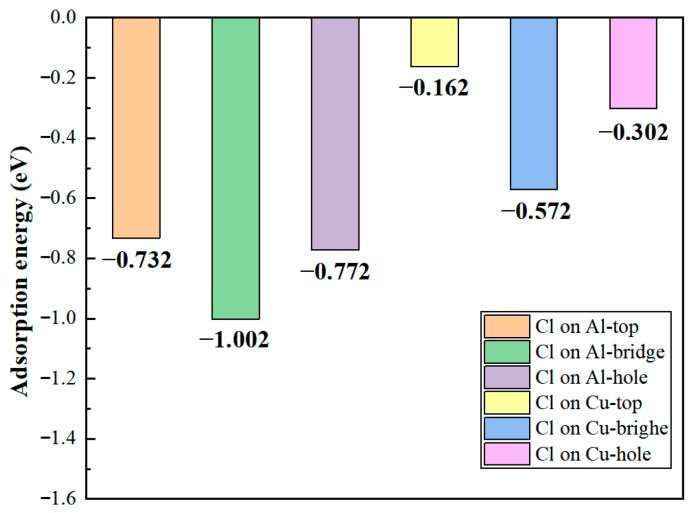
Adsorption energies of Cl^−^ on different adsorption sites on the Al_2_Cu(110) surface.

The adsorption energies of Cl^−^ at various sites on the structurally optimized Al_2_Cu(110) surface model were calculated by using Equation (16), as shown in Figure 4. Both the type of substrate atom and the geometric configuration of the adsorption site significantly influence the adsorption stability of Cl^−^. On Al substrate sites, the adsorption energies are generally more negative compared to those on Cu substrate sites. This suggests that Cl^−^ binds more strongly to Al atoms, leading to more stable adsorption systems. Within the same metal substrate, the absolute values of the adsorption energies follow the order: bridge site > hole site > top site. From a thermodynamic perspective, the more negative adsorption energies on Al compared to Cu sites reflect a reduced Gibbs free energy of adsorption, indicating a stronger driving force for Cl^−^ to bind to Al atoms and stabilize configurations. This aligns with the quantitative correlation between adsorption energy and thermodynamic stability, with more negative values indicating greater stability.

The microscopic mechanism underlying these differing adsorption energies can be elucidated from two aspects. First aspect is the inherently different electronic structures. The difference between the electronegativities of Al (1.61) and Cl^−^ (3.16) is larger than that between Cu (1.90) and Cl^−^ [50]. This larger difference drives significant charge transfer from Al to Cl, inducing the formation of an Al–Cl bond characterized by a stronger ionic character. By contrast, the fully occupied 3d orbitals of Cu enhance covalent interactions with Cl^−^. However, the Cl^−^ ligand exhibits a weak π-accepting capability, resulting in a limited contribution from back-donated π bonding and consequently weaker overall bond strength. The second aspect involves site geometry and coordination effects. The Al-bridge site forms bonds with two Al atoms through bidentate adsorption, where orbital overlap and electrostatic interactions synergistically enhance stability, resulting in the most stable configuration. Conversely, the Al-top site exhibits a monodentate adsorption mode characterized by single-dimension bonding, leading to the lowest adsorption stability. The Al-hole site demonstrates intermediate stability due to the restricted bonding symmetry caused by its multi-coordination geometry. Although a similar trend is observed for the Cu substrate sites, the absolute adsorption energies of these sites are generally lower due to the inherently weaker Cu–Cl bonds.

**Figure 5:**
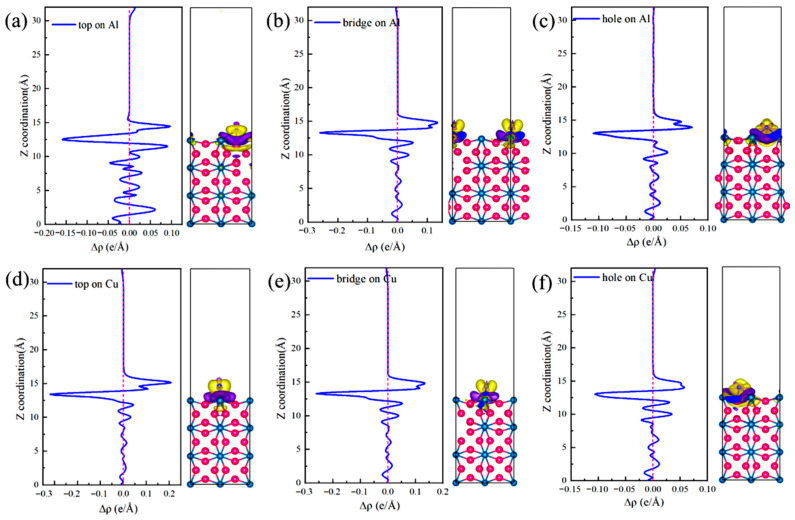
Differential charge densities of Cl^−^ adsorbed on the Al_2_Cu(110) surface at different sites: (**a**) Al-top, (**b**) Al-bridge, (**c**) Al-hole, (**d**) Cu-top, (**e**) Cu-bridge, and (**f**) Cu-hole.

**Figure 6:**
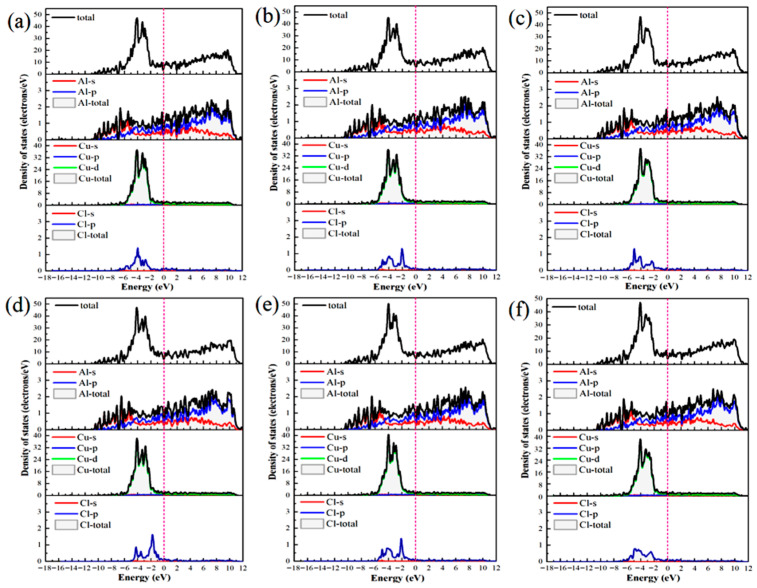
Density of states for Cl^−^ adsorbed on the Al_2_Cu(110) surface at different sites: (**a**) Al-top, (**b**) Al-bridge, (**c**) Al-hole, (**d**) Cu-top, (**e**) Cu-bridge, and (**f**) Cu-hole.

The differential charge density (Figure 5) and DOS (Figure 6) were analyzed to elucidate the atomic-scale origins of these adsorption energy differences, revealing fundamental distinctions in the bonding modes between Al and Cu sites. The core mechanism elucidating these differences involves competition between strongly localized ionic bonds and covalent interactions. In chemical bond theory, the essential difference between σ and π bonds lies not only in the patterns of orbital overlap but also in the localization and delocalization characteristics of the electron clouds [51,52]. Notably, σ bonds are formed through “head-to-head” overlap, where electron clouds are highly concentrated along the bond axis, leading to strong localization. By contrast, π bonds are formed through “shoulder-to-shoulder” overlap involving the distribution of electron clouds on both sides of the bond axis, which leads to delocalization. Moreover, electrons can be shared among multiple atoms, in particular, in multi-center delocalized π bonds. In surface adsorption, this difference in localization and delocalization directly influences charge transfer and spatial distribution patterns, thus aiding in determining the tendency toward ionic or covalent bonding.

Adsorption interactions at the Al sites are primarily characterized by hybrid bonding, which is predominantly influenced by s–p orbitals and exhibits strong ionic bond characteristics. Differential charge density analysis (Figure 5a–c) indicates that at the Al-top site, the 3p orbital of Cl^−^ undergoes hybridization with the 3p orbital of Al, resulting in significant localized electron transfer from Al atom to Cl^−^. This phenomenon creates highly directional and strongly localized bonding, with nuclear enrichment along the bond axis. Substantial electron accumulation is observed directly above Cl^−^ (yellow region), and correspondingly, pronounced electron depletion occurs around the Al atom (purple region), forming a sharp charge dipole that is perpendicular to the surface. The concentrated charge transfer along the bond axis at this site is characteristic of “head-to-head” orbital overlap, which facilitates the formation of strongly localized σ bonds and minimizes the energy penalty for Cl^−^ to stay adsorbed. At the Al-bridge site, the 3p orbital of Cl^−^ forms σ bonds with two adjacent Al atoms and also establishes weaker π interactions via lateral overlap. This σ–π hybrid bonding leads to a more complex charge distribution in which the electron density around the Cl^−^ exhibits heterogeneous patterns of accumulation and dissipation. At the Al-hole site, the Cl^−^ interacts with multiple surrounding Al atoms, resulting in extensive orbital overlap and a broader charge distribution. The electron cloud surrounding the Cl^−^ shows a relatively uniform distribution with some directional variations, suggesting a delocalized yet robust binding network that kinetically traps Cl^−^ through interactions involving distributed charges. Projected density of states (PDOS) analysis (Figure 6a–c) highlights distinct hybridization peaks between Cl-3p and Al-3p orbitals at deeper energy levels, focusing on the orbitals of the adsorbed Cl^−^ and interacting surface Al atoms. Combined with localized charge transfer observed in the system, these results suggest that the Al–Cl bond is an ionic–covalent hybrid bond with notable charge transfer. Kinetically, adsorption of Cl^−^ at Al sites is kinetically preferred, attributed to lower adsorption activation barriers and enhanced dynamic stability of the adsorbed state. This reduces the energy required for Cl^−^ to overcome initial surface reconstruction barriers during attachment. Moreover, directional charge transfer forms a potential well that enhances resistance to desorption. This microscopic mechanism helps explain the relatively high bond strength and more negative adsorption energies associated with Al–Cl bonds at these adsorption sites.

At the Cu sites, the d-orbital of Cu results in distinct hybridization modes. Differential charge density analysis (Figure 5d–f) indicates the spatial dispersion of charge transfer at the Cu-top site. A large electron depletion region is observed around the Cu atom, and an extensive electron accumulation region is located near Cl^−^. This dispersed charge distribution aligns with the delocalization resulting from “shoulder-to-shoulder” orbital overlap. At the Cu-bridge site, the symmetrical coordination environment facilitates uniform charge transfer between the two Cu atoms, leading to a highly symmetric charge distribution. Furthermore, at the Cu-hole site, the electronic structure surrounding the defect diminishes the efficiency of orbital overlap between Cu and Cl^−^, resulting in weak bonding signals and gradual changes in charge density. The PDOS analysis (Figure 6d–f) indicates that the Cu-3d orbitals dominate the electronic states near the Fermi level (E_F_), with a minor contribution from Cl-3p states and limited orbital hybridization between them. This electronic structure suggests a weak interaction between d–p orbitals and delocalized charge transfer at these sites, consistent with their lower Cu–Cl bond strength and smaller absolute adsorption energies observed (Figure 4).

Briefly, the strong ionic bonding between Al sites and Cl^−^ is characterized by highly localized charge transfer and highly directional electron concentration. By contrast, the interaction between Cu sites and Cl^−^ is characterized by a relatively diffuse charge distribution and weaker orbital hybridization, with covalent interactions being more prominent. This competition between localized ionicity and delocalized covalency constitutes the fundamental electronic structural origin of the significant variations in the adsorption energies of Cl^−^ at different sites on the Al_2_Cu surface.

**Figure 7:**
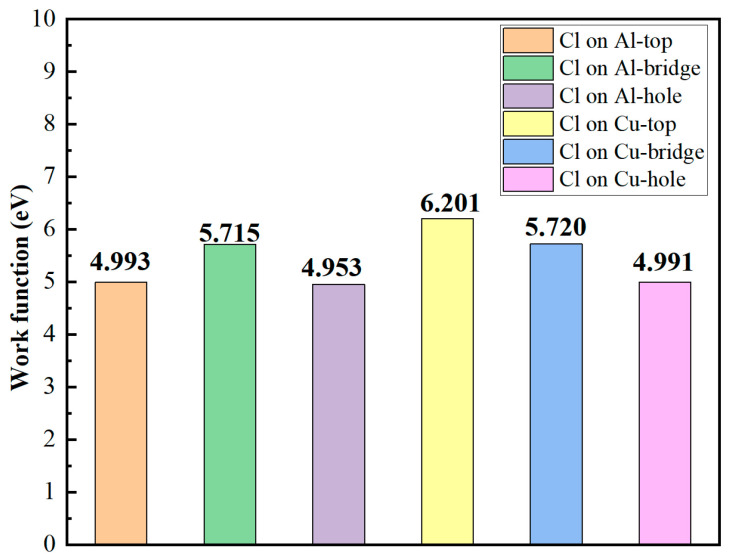
Electron work functions of Cl^−^ adsorption at different sites on the Al_2_Cu(110) surface.

The work function, which is a key parameter that directly reflects the difficulty of electron escape [53,54], was also utilized to characterize the adsorption behavior of chlorine on the different sites of the Al_2_Cu(110) surface, as shown in Figure 7. All adsorption sites show a notable increase in the adsorption-induced surface work function compared to the unabsorbed state, with the magnitude of the work function following the order: unadsorbed < Al-hole < Cu-hole < Al-top < Al-bridge < Cu-bridge < Cu-top. This increasing trend is attributed to the highly electronegative chlorine atoms that extract electrons from the surface, which results in the formation of an outward-facing surface dipole layer. The presence of this dipole layer provides an additional internal electric field, which raises the potential barrier for electron escape.

Notably, although the Al–Cl bonds exhibit stronger ionic characteristics, indicating greater charge transfer, the Cu sites show a more pronounced enhancement in the work function, with the Cu-top configuration exhibiting the highest value. This observation suggests that instead of solely being influenced by the total charge transfer, variations in the work function are also critically affected by the strength of the surface dipole moment. The intensity of the dipole moment is jointly determined by the effective charge transfer and the dipole arm length, both of which are precisely regulated by the local geometry and electronic structure of each adsorption site [55]. The adsorbed Cl^−^ on the Cu-top site is directly exposed to the vacuum, serving as a single-atom coordination site. This Cl^−^ maintains the maximum vertical distance from the charge center of the substrate, forming the longest dipole arm. Furthermore, this site lacks neighboring atoms for electronic shielding, which enables the localized dipole field to interact most efficiently with the surface. Consequently, the Cu-top configuration, with its longest dipole arm and minimal shielding effect, demonstrates the most significant increase in the work function, leading to the most substantial enhancement of the surface dipole moments.

Despite the distinct intrinsic bonding characteristics of Al and Cu, the work functions of the Al-bridge and Cu-bridge sites are comparably high. This observation indicates that the bidentate coordination mode of the bridge adsorption sites provides a pronounced and stable dipole effect. The Al-hole and Cu-hole sites also exhibit similar work functions, which is likely due to their analogous geometries and coordination environments. These factors influence the adsorption configuration and charge redistribution of Cl^−^, thereby mitigating the inherent differences between the metals. High work function values suggest stronger electronic confinement and a more inert surface. Therefore, adsorption sites with higher work functions could potentially serve as passivation centers. Conversely, sites with low work functions consist of more readily accessible electrons, resulting in heightened electrochemical reactivity.

In summary, the adsorption energy, electronic structure, and work function values of Cl^−^ adsorbed on different sites on an Al_2_Cu surface were comprehensively analyzed to elucidate the microscopic mechanism by which chloride adsorption influences the initial pitting nucleation in Al_2_Cu alloys. Cl^−^ exhibits a strong preference for adsorption onto the Al sites, both thermodynamically and kinetically, resulting in enhanced adsorption stability. This adsorption process weakens the bonds between the surface Al atoms and reduces their electronic binding energy, significantly lowering the energy barrier for anodic dissolution in electrochemical environments. Consequently, pitting nuclei are expected to predominantly form at these Al-rich active sites. By contrast, Cl^−^ adsorption on Cu sites, in particular, in the Cu-top configuration, leads to a substantial increase in the work function and surface passivation, which hinders oxidation dissolution. Thus, Cu-rich phases likely exhibit stable cathodic behavior during initial corrosion processes. The pronounced disparity in the electrochemical activities of Al-rich and Cu-rich regions creates a synergistic effect. That is, the rapid dissolution of active Al sites combined with the occurrence of cathodic reactions at Cu sites induces the formation of an efficient micro-coupled corrosion cell, dramatically accelerating the deepening of localized corrosion in Al-rich zones.

### 3.2. Adsorption Properties of Cl^−^ at the Al(111)/Al_2_Cu(110) Interface

**Figure 8:**
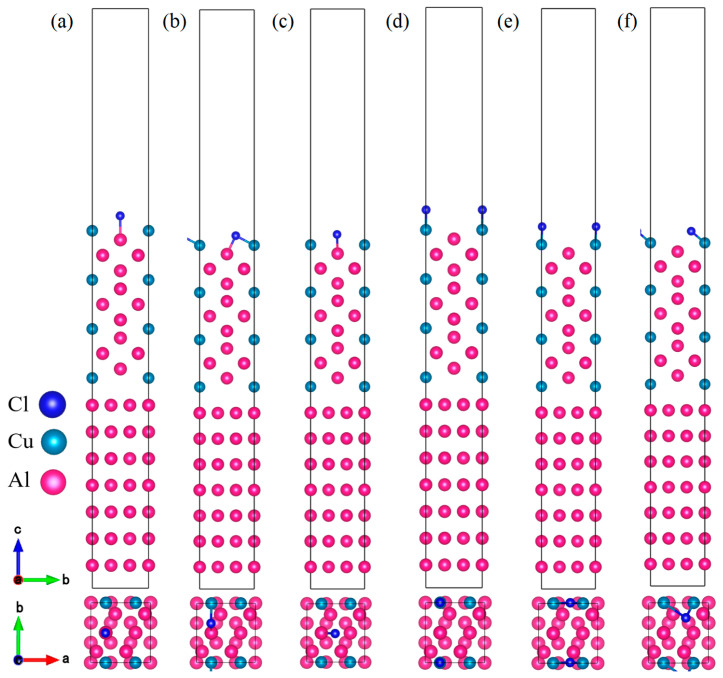
Atomic models of Cl^−^ adsorption on the Al(111)/Al_2_Cu(110) interface: (**a**) Al-top site, (**b**) Al-bridge site, (**c**) Al-hole site, (**d**) Cu-top site, (**e**) Cu-bridge site, and (**f**) Cu-hole site.

In the casting–rolling production of Cu–Al layered composites, an Al_2_Cu intermetallic layer is formed at the interface. Besides serving as a key load-carrying constituent, this intermetallic layer acts as a preferred channel for corrosive ingress, making it a preferential site for corrosion initiation [56]. Based on this understanding, an adsorption model covering six characteristic sites (Al-top, Al-bridge, Al-hole, Cu-top, Cu-bridge, Cu-hole) was constructed at the Al(111)/Al_2_Cu(110) interface (Figure 8). Subsequently, the interfacial adsorption behavior of Cl^−^ on this interface was compared with that on a single Al_2_Cu(110) surface to clarify the mechanism by which the interfacial architecture influences the Cl^−^ adsorption modes and the role of this interface in triggering corrosion.

**Figure 9:**
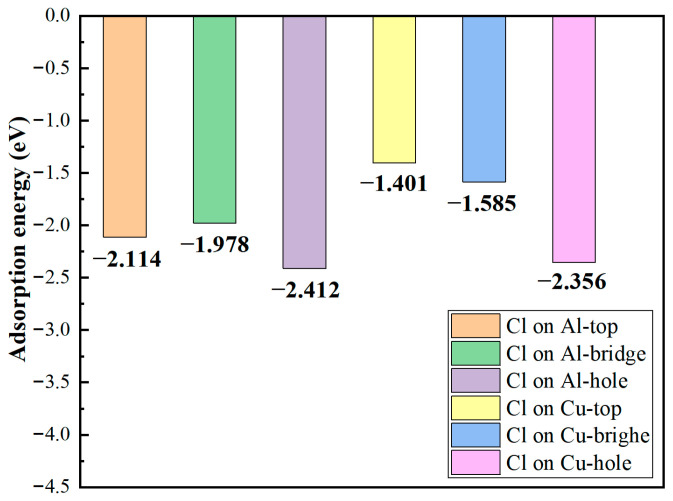
Adsorption energies of Cl^−^ at different sites on the Al(111)/Al_2_Cu(110) interface.

First, interfacial adsorption stability was evaluated by determining the adsorption energies of Cl^−^ at the Al(111)/Al_2_Cu(110) interface. Figure 9 illustrates that Cl^−^ exhibits significant site-dependent adsorption energies at different interface adsorption sites. Overall, the adsorption energies of this interface system are generally more negative than those of the corresponding single Al_2_Cu(110) surface. Therefore, from a thermodynamic perspective, Cl^−^ is more stably adsorbed at this heterogeneous interface, which acts as an efficient “trap” for Cl^−^. This finding directly confirms the driving force behind the preferential initiation of pitting nuclei at the interface in the initial corrosion stage.

Within the Al(111)/Al_2_Cu(110) interface, the intrinsic differences between Al and Cu influence the stability of Cl^−^ adsorption, similar to the trend observed on the single Al_2_Cu(110) surface. Specifically, the Al substrate sites exhibit more negative adsorption energies and higher stability compared to the Cu substrate sites. This confirms that the strong polar/ionic bonding strength between Al and Cl^−^ far surpasses that of the Cu–Cl system. However, unlike the varying adsorption energy trends observed at different sites on the single Al_2_Cu(110) surface, the most stable site at the Al(111)/Al_2_Cu(110) interface shifts to the Al-hole position, followed by the Cu-hole configuration. This transformation is attributed to the unique atomic stacking and coordination environment at the interface. The increased density of Al atoms at this interface allows the hole positions formed by multiple Al atoms to provide superior multi-coordination geometry, enabling efficient orbital overlap and charge transfer between Cl^−^ and adjacent Al atoms. Consequently, the adsorption system stability is maximized. By contrast, although the interface bridge site maintains double coordination, the bonding at this site may be modulated by lattice mismatch and stress fields, preventing optimal adsorption stability.

Table 1 provides a comparison of the work functions of Cl^−^ adsorbed on the single Al_2_Cu(110) surface with those of the Al(111)/Al_2_Cu(110) interface. In general, adsorption sites on this interface system exhibit lower work function values than the corresponding sites on the single Al_2_Cu(110) surface. This phenomenon suggests that the formation of the Al(111)/Al_2_Cu(110) heterointerface introduces lattice mismatch and charge redistribution, collectively diminishing the surface dipole effect induced by the adsorption of Cl^−^.

Nevertheless, the Cu-top site maintains the highest work function value in the interfacial region. Although this work function is slightly lower than that of the Cu-top site on the single Al_2_Cu(110) surface, it remains significantly higher than that of the other interface sites. This indicates that, even in the interface environment, the Cu-top site can form a strong localized dipole layer due to its unique single-atom coordination and potential d–p orbital interactions, thereby maintaining high surface inertness. Conversely, the work function of the Al-bridge site at the interface is significantly lower than that of the corresponding surface site. This suggests that lattice relaxation and electronic structure adjustments at the interface weaken the dipole moment of this site, reducing the electron escape energy and potentially enhancing its electrochemical activity.

**Figure 10:**
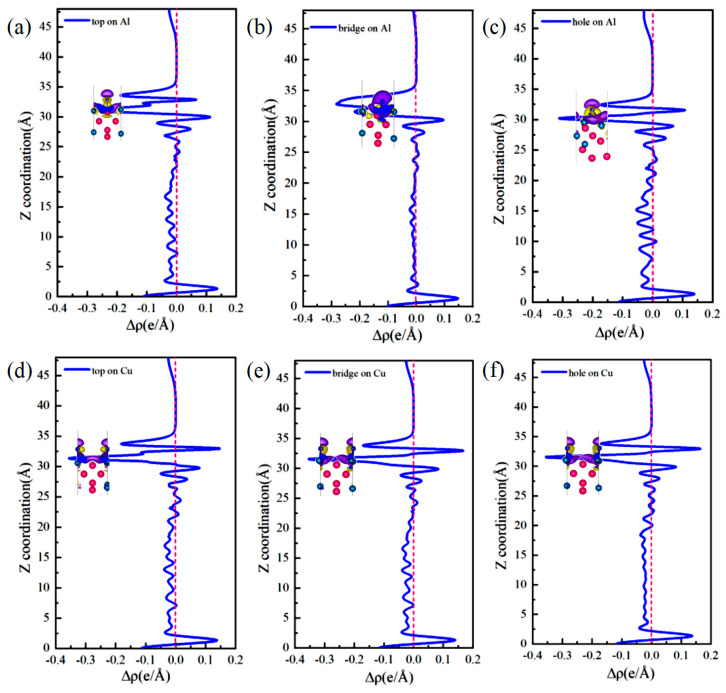
Differential charge densities of Cl^−^ adsorbed on the Al(111)/Al_2_Cu(110) interface at different sites: (**a**) Al-top, (**b**) Al-bridge, (**c**) Al-hole, (**d**) Cu-top, (**e**) Cu-bridge, and (**f**) Cu-hole.

**Figure 11:**
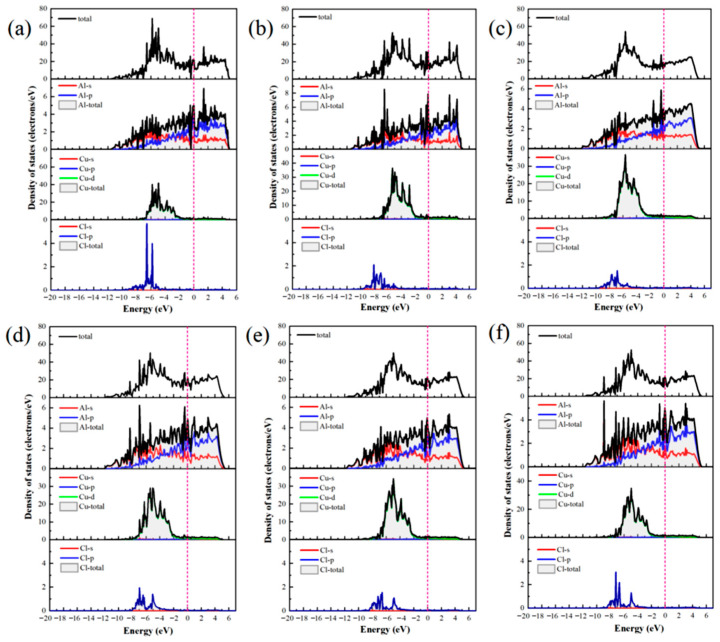
Density of states for Cl^−^ adsorbed on the Al(111)/Al_2_Cu(110) interface at different sites: (**a**) Al-top, (**b**) Al-bridge, (**c**) Al-hole, (**d**) Cu-top, (**e**) Cu-bridge, and (**f**) Cu-hole.

The differential charge densities (Figure 10) and DOS (Figure 11) of the six interface adsorption sites distinctly illustrate the influence of the interface environment on adsorption bonding at the electronic level. Compared to the single Al_2_Cu(110) surface, the Al(111)/Al_2_Cu(110) interface shows notably more pronounced charge density oscillations, indicating that interfacial coupling leads to a more complex charge redistribution. For example, at the Al-bridge site of the interface (Figure 10b), the purple charge depletion region extends significantly beyond the *Z*-axis direction (surface normal) compared to that of the corresponding surface site. This observation clearly demonstrates that the interface structure facilitates electron transfer from broader substrate regions to the adsorbed Cl atoms, leading to enhanced charge delocalization. At the Al-bridge site (Figure 11b), PDOS analysis of the adsorbed Cl^−^ and the surface Al atoms reveals that the hybridization peaks of Cl-3p and Al-3p orbitals below E_F_ become broader and shift to deeper energy regions, which aligns with the previous observation.

On the single Al_2_Cu(110) surface, Al sites exhibit clear hybridization between Cl-3p and Al-3p orbitals at relatively deep energy levels, corresponding to ionic-dominant bonding with significant charge transfer. The Cu sites exhibit weak overlap between Cl-3p and Cu-3d orbitals near E_F_, indicating relatively weak d–p back-bonding character. At the interface, however, the bonding characteristics exhibit some moderate changes. Specifically, for the Al sites (Figure 11a–c), the hybridization peaks of Cl-3p and Al-3p orbitals generally shift toward deeper energy regions, along with an increase in peak intensity in the PDOS. This phenomenon illustrates the intensified orbital hybridization between Al and Cl^−^ at the interface, indicating a significant increase in the covalent component of the chemical bond. This can be fundamentally explained by the introduction of densely packed Al(111) planes at the interface, which establishes geometric and electronic structural synergies with the Al atoms in Al_2_Cu at the atomic scale. Furthermore, modulation of the spatial distribution and orientation of Al-3p orbitals promotes more effective hybridization with Cl-3p orbitals. This enhanced interaction is directly revealed by the PDOS for the adsorbed Cl^−^ and interfacial Al atoms, strengthening both σ-bond coupling and charge transfer driven by the differences in electronegativity. The intensified orbital hybridization and Coulombic attraction work together to facilitate the formation of a polar σ bond at the interface. Although the bond remains predominantly ionic in character, its overall strength increases by enhanced orbital overlap and electrostatic interactions. These electronic variations are clearly manifested in the PDOS as a downward shift and broadening of the Cl-3p and Al-3p hybridization peaks toward deeper energy levels.

At the Cu sites (Figure 11d–f), the hybridization peaks of the Cl-3p and Cu-3d orbitals exhibit a negative energy shift, although the magnitude of this shift is generally smaller than that observed at the Al sites. This phenomenon is attributed to the presence of Al(111) layer at the interface, which modulates the orbitals of the Cu atoms in Al_2_Cu. On the one hand, the presence of the Al(111) layer slightly suppresses the spatial extension and electronic activity of the Cu-3d orbitals, reducing their already weak covalent contribution to the Cl-3p orbitals. On the other hand, the charge redistribution and built-in electric field induced by the formation of the interfacial heterostructure intensify the electrostatic attraction between the Cl^−^ and Cu atoms through charge polarization. Consequently, bond polarity enhances and the ionic character of the bond becomes more prominent compared to the single-surface environment. In the DOS maps, although the hybridization peaks of the Cu–Cl system show an overall shift toward deeper energy levels, the magnitude of this shift is limited, and relatively small changes in the peak shape are observed. This reflects a compositional rebalancing of the bonding nature at the interface, i.e., on the single Al_2_Cu(110) surface, Cu–Cl bonds are characterized by weak covalent feedback with low polarity. Moreover, at the interface, both covalence and polarity remain low, but the proportion of the polar contribution increases. Noteworthy, this transformation does not fundamentally alter the basic qualitative nature of the Cu–Cl bond, which remains weakly covalent and dominated by covalent interactions. Nevertheless, this change reveals how the unique physicochemical environment at the interface adjusts the bond composition by suppressing covalent feedback and enhancing electrostatic attraction, which affects the Cl adsorption stability and surface reactivity of the Cu sites.

Integrated thermodynamic and electronic structure analyses reveal that the heterogeneous Al(111)/Al_2_Cu(110) interface primarily functions as both a “Cl^−^ enrichment zone” and an electrochemical “active source” to synergistically reshape corrosion initiation pathways. The higher adsorption energies of the interface sites indicate the strong capability of the interface for capturing Cl^−^, which promotes Cl^−^ accumulation in the interface region. Specifically, the Al-hole adsorption site exhibits the highest stability, serving as the preferred nucleation site for pitting nuclei. Moreover, the Al-top and Al-bridge sites, which are activated by their lower work functions, collectively form an extended anodic active region. Cl^−^ adsorption at these sites triggers significant charge transfer, which weakens the surface Al metal bonds and lowers the dissolution energy barrier. Concurrently, the Cu-top site maintains a high adsorption energy and work function, thus preserving its inert cathodic role. This creates a spatially adjacent yet functionally distinct pairing of the “active Al region–inert Cu spot” at the interface. These conditions collectively establish an efficient micro-couple corrosion cell: Cl^−^ enrichment sustains continuous corrosion reactions, the expanded anodic region provides additional dissolution sites, and the adjacent cathodic region efficiently maintains cathodic reactions. This tripartite mechanism synergistically influences both the thermodynamics and kinetics of the corrosion reaction. Pitting nuclei are thermodynamically driven to preferentially nucleate at the interface, and the dissolution of Al-rich phases is kinetically accelerated through enhanced micro-couple effects. Thus, rapid inward corrosion propagates along the interface.

### 3.3. Fe–Si Doping Effects on Al/Al_2_Cu Interface Stability and Cl^−^ Adsorption

**Figure 12:**
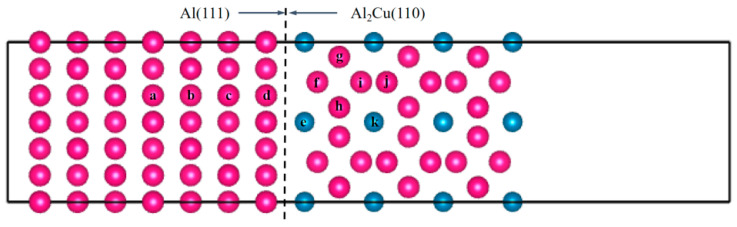
Atomic model of Al(111)/Al_2_Cu(110) interface and atomic substitution positions.

To elucidate the influence of Fe or Si micro-alloying on Cl^−^ adsorption and corrosion initiation at the Al/Al_2_Cu interface, first-principles calculations based on the established Al(111)/Al_2_Cu(110) coherent interface model were employed in this study. Single-atom substitutions of Fe or Si were carried out at 11 distinct interfacial sites (labeled as a–k, Figure 12) to model solute segregation. The computational slab comprised seven Al layers and 13 Al_2_Cu layers, with the bottom three atomic layers fixed and all others fully relaxed. A plane-wave cutoff of 500 eV and a 4 × 4 × 1 k-point mesh were used. Further, the substitution energy (ΔE_sub_), adhesion work (W_ad_), and interfacial energy (γ_int_) were systematically calculated for each doped configuration to assess their thermodynamic stability and interfacial cohesion.

#### 3.3.1. Selection of Fe and Si Atom Doping Sites

**Figure 13:**
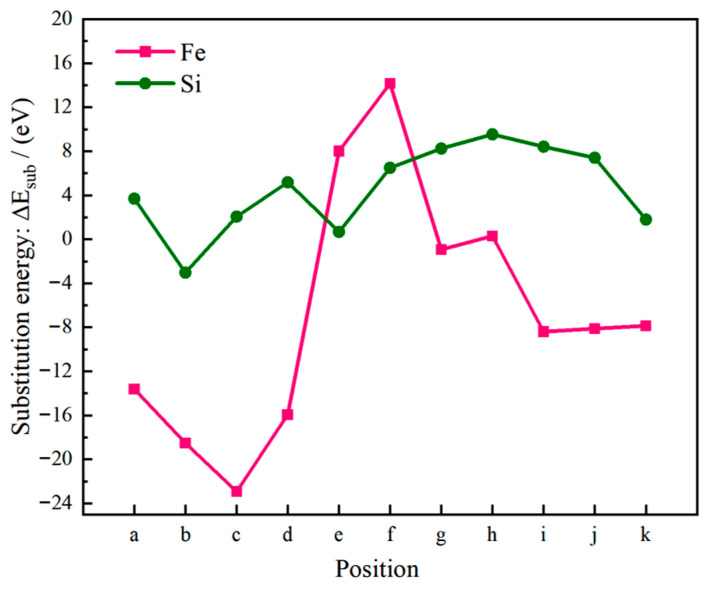
Substitution energies of Fe and Si in the Al(111)/Al_2_Cu(110) interface model.

**Figure 14:**
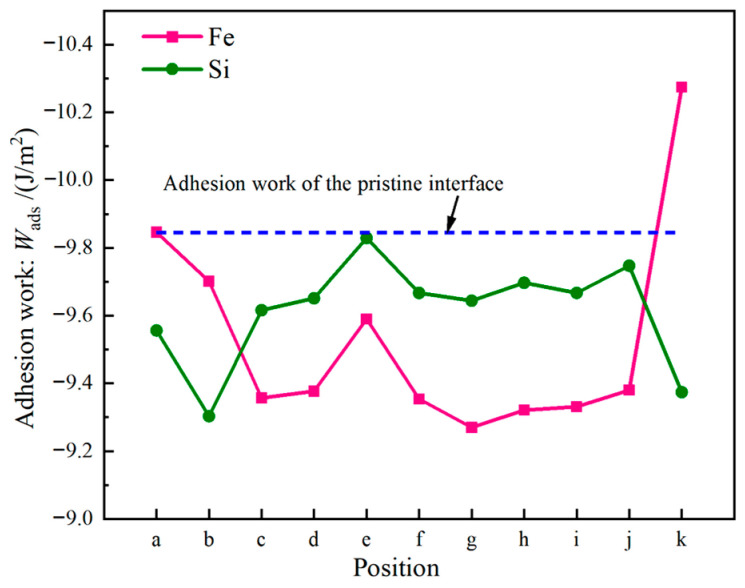
Adhesion work of Fe and Si in the Al(111)/Al_2_Cu(110) interface model.

**Figure 15:**
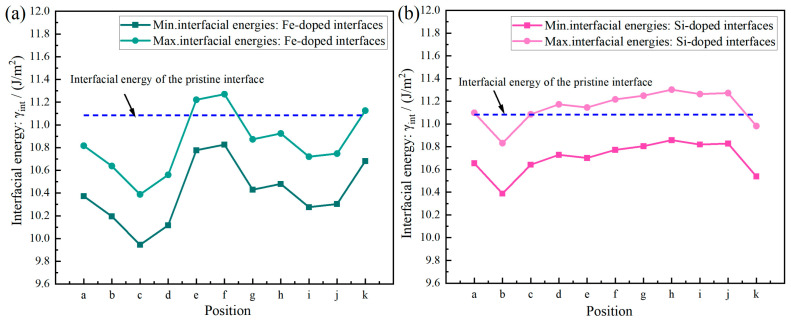
Interfacial energies of Fe/Si-doped Al(111)/Al_2_Cu(110) interfaces: (**a**) Fe-doped interface; (**b**) Si-doped interface.

Based on Formulas (12)–(15), the surface energy, substitution energy, adhesion work, and interfacial energy values obtained after doping Fe or Si atoms at 11 characteristic positions were calculated, as illustrated in Figure 13, Figure 14 and Figure 15. A more negative substitution energy (ΔE_sub_) indicates that atomic substitution is more thermodynamically favorable [57]. Si exhibits consistently low and stable substitution energies, suggesting that substrate atoms on both sides of the Al(111)/Al_2_Cu(110) interface can be easily replaced by Si atoms with a minimal energy cost. This highlights the favorable solid solubility of Si in this system, which is attributed to the strong compatibility between Si and Al in terms of atomic radius and electronegativity. Consequently, the introduction of Si results in limited lattice distortion and enhanced energetic stability, which promotes solid solution formation. By contrast, the substitution energy of Fe shows considerable variation across different sites. For instance, the c-site (on the Al side) shows a very low substitution energy, indicating a strong tendency for Fe to replace Al, whereas the f-site (on the Al_2_Cu side) exhibits a significantly higher substitution energy, reflecting a much weaker substitution tendency. This pronounced difference stems from the distinct crystallographic environment of Al_2_Cu, which is a tetragonal IMC with a closely packed atomic arrangement. Si and Fe atoms exhibit significant differences in terms of both atomic size and electronegativity, leading to poorer compatibility with the Al/Cu sublattices in the Al_2_Cu phase. Consequently, a substantial lattice distortion energy must be overcome to substitute Fe into Al_2_Cu, making this substitution less favorable. By contrast, substitution on the Al side proceeds more readily due to the greater similarities in atomic size and electronegativity between Fe and Al.

The magnitude of the adhesion work (*W*_ads_) reflects the strength of interfacial bonding, with a larger value signifying stronger cohesion [58]. Figure 13, Figure 14 and Figure 15 illustrate that Si doping yields substantially higher *W*_ads_ values than Fe doping, indicating that the introduction of Si markedly enhances the interfacial adhesive strength. This improvement is attributed to the predominantly covalent or semi-covalent character of the bonds formed by Si with Al and Cu. Owing to the moderate electronegativity of Si, the electron clouds of Si atoms can efficiently overlap with those of neighboring atoms. Thus, bonding across both sides of the interface is accommodated, which leads to significant energy stabilization and robust interfacial cohesion. Conversely, Fe generally exhibits lower *W*_ads_ values, with only isolated cases approaching those of Si. Fe displays the most favorable substitution energy on the Al side (e.g., the c-site), indicating that Fe can be readily incorporated into the Al lattice. However, the resulting Fe–Al bonds are characterized by strong polar metallic bonding with limited orbital hybridization, indicating that the *W*_ads_ values are only marginally improved relative to the undoped interface. Conversely, the highest Fe substitution energies are observed on the Al_2_Cu side (e.g., the f-site), implying that Fe cannot easily enter the intermetallic lattice. Consequently, these sites show the lowest *W*_ads_ values, revealing that Fe neither integrates effectively into Al_2_Cu nor contributes to durable interfacial bonding. Eventually, this leads to poor interfacial stability.

Interfacial energy (*γ*_int_) reflects the combined effects of atomic misfit and bonding coordination, and a lower *γ*_int_ value corresponds to a more thermodynamically stable interface. Overall, the Si-doped interfaces exhibit lower *γ*_int_ values compared to the Fe-doped interfaces, indicating that the addition of Si provides superior interfacial stability. This can be partially ascribed to the similar atomic radii of Si and Al leading to greater compatibility. Moreover, the propensity of Si to form predominantly covalent bonds with Al/Cu enhances structural compatibility across the interface. Consequently, incorporating Si reduces both the misfit strain and bonding defects among the interfacial atoms, leading to a decreased *γ*_int_ and improved stability. By contrast, Fe doping leads to significant fluctuations in interfacial energy with local maxima. At the c-site (Al side), Fe substitution is the most energetically favorable, with a moderately lower *γ*_int_ value for Fe doping compared to Si doping, despite the accompanying lower adhesion work. This suggests that although Fe can occupy Al lattice sites, the resulting strongly polar Fe–Al bonds do not substantially enhance the interface. Furthermore, the electrochemical contrast between Fe (acting as a local anode) and Al (acting as a cathode) introduces a tangible risk of galvanic corrosion. Moreover, the f-site (Al_2_Cu side) exhibits the highest Fe substitution energy, indicating that substitution at this location is extremely challenging. Therefore, this site exhibits the highest *γ*_int_. This analysis confirms that Fe cannot be effectively integrated into the Al_2_Cu lattice, with significant atomic misfit and poor bonding leading to a highly unstable interfacial configuration.

The substitution energy quantifies the thermodynamic tendency for atomic incorporation into the interfacial lattice, whereas the adhesion work and interfacial energy govern the resulting bond strength and structural stability after incorporation. Specifically, the substitution energy values indicate that Si has low substitution energy, making its incorporation thermodynamically favorable. Moreover, the adhesion work and interfacial energy together demonstrate that incorporation of Si enhances structural stability, reinforces cohesive bonding, and enhances interfacial integrity. Together, these factors support the formation of extensive solid solutions across both sides of the Al(111)/Al_2_Cu(110) interface. Consequently, Si effectively strengthens the interface. By contrast, Fe can only readily form a solid solution on the Al side (e.g., at the c-site), which carries a concomitant risk of galvanic corrosion. On the Al_2_Cu side (e.g., the f-site), Fe shows a high substitution energy, poor solubility, and weak interfacial bonding, indicating that it is not suitable for interfacial reinforcement.

#### 3.3.2. Adsorption Properties of Cl on Al(111)-Fe-Si/Al_2_Cu(110) Surfaces

**Figure 16:**
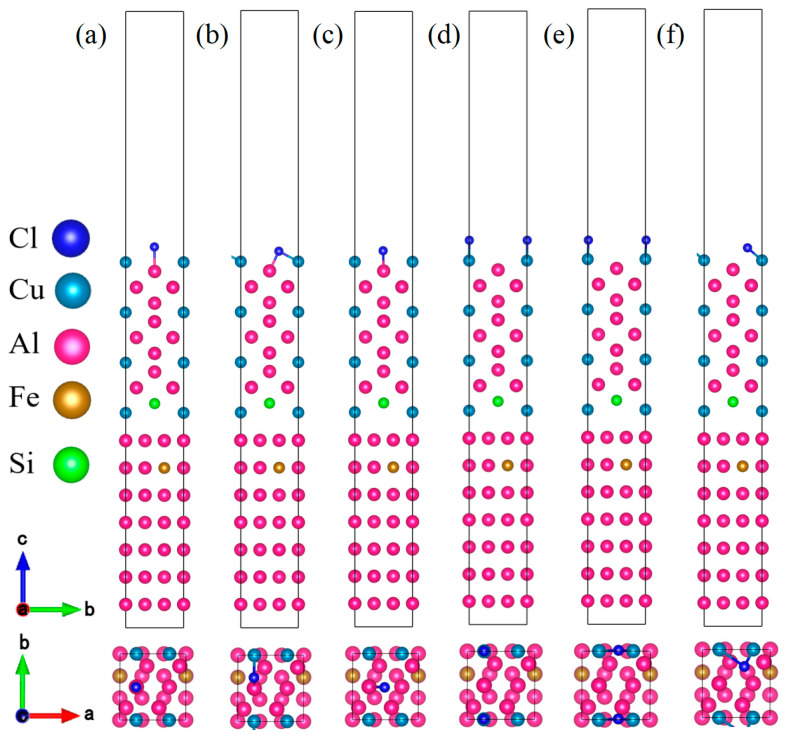
Atomic models of Cl^–^ adsorption on the Al(111)-Fe-Si/Al_2_Cu(110) interface: (**a**) Al-top site, (**b**) Al-bridge site, (**c**) Al-hole site, (**d**) Cu-top site, (**e**) Cu-bridge site, and (**f**) Cu-hole site.

**Figure 17:**
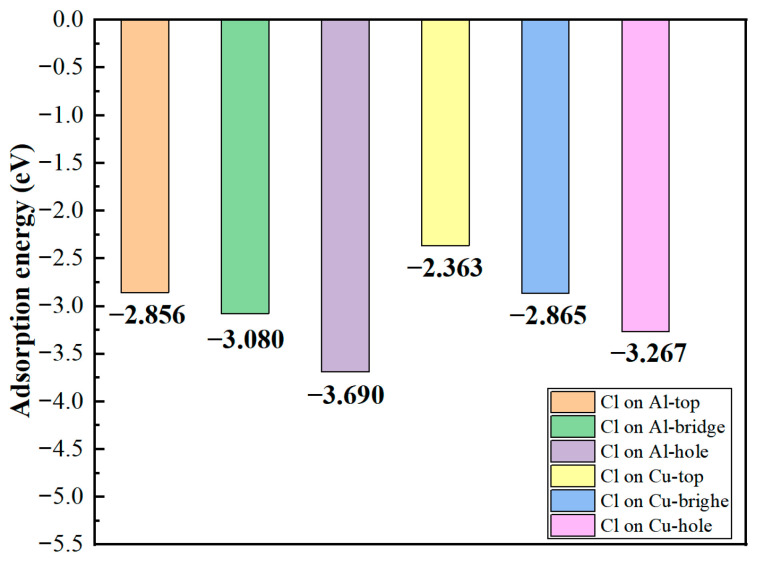
Adsorption energies of Cl^−^ at different sites on Al(111)-Fe-Si/Al_2_Cu(110) interface.

After identifying the optimal doping sites for Fe and Si atoms, an adsorption model was constructed (Figure 16) to investigate the microscopic mechanisms by which Fe/Si substitution modulates the Cl^−^ adsorption behavior at the Al(111)-Fe-Si/Al_2_Cu(110) interface. The calculated absolute values of Cl^−^ adsorption energy at the Fe–Si-doped interface are significantly higher than those at the undoped Al(111)/Al_2_Cu(110) interface (Figure 17). Therefore, Fe and Si doping substantially enhances the interfacial adsorption stability of Cl^−^, providing a stronger thermodynamic driving force for the sustained accumulation of chloride at the interface. This enhanced adsorption stability arises from the synergistic effects of electronic structure reconstruction and local coordination environment optimization induced by Fe–Si doping. The electronic structures of both Fe and Si are distinct from those of Al and Cu, and the Fe and Si dopants form characteristic chemical bonds with neighboring atoms upon doping, which modulates the interfacial charge distribution. Specifically, Si atoms readily form strong covalent or semi-covalent bonds with Al and Cu at the interface due to their moderate electronegativity and favorable size match with Al. This enhances charge delocalization and orbital overlap, increasing the electron density available at potential Cl^−^ adsorption sites. Moreover, the partially filled 3d orbitals of the Fe atoms substituted on the Al side can lead to the formation of polar metallic bonds with Al sp-orbitals, further tuning the interfacial charge distribution and collectively strengthening the electrostatic and orbital interactions between Cl^−^ and the interface.

Moreover, Fe and Si doping optimizes the geometric arrangement and coordination symmetry of the interfacial atoms, providing more stable and electronically favorable sites for Cl^−^ adsorption. Notably, the introduction of Si at weakly bonded sites mitigates local lattice distortion and improves orbital matching and overlap efficiency. Concurrently, the incorporation of Fe at multi-coordinated sites, such as the Al-bridge site, enhances the coordination of adsorbed Cl^−^ and stabilizes the adsorption structure through lattice strain effects, rendering the overall configuration more energetically favorable.

This pronounced enhancement in Cl^−^ adsorption stability on the Al(111)-Fe-Si/Al_2_Cu(110) interface presents significant implications for practical corrosion scenarios involving layered Cu–Al composites. The presence of Fe and Si dopants promotes the sustained adsorption and accumulation of Cl^−^ at interfacial active sites, creating localized zones with high Cl^−^ concentrations. These dopants also undermine the stability of the surface passive film, leading to the more rapid nucleation and propagation of localized attacks. Furthermore, the enhanced adsorption of Cl^−^ facilitates inward diffusion of Cl^−^ along interfaces or grain boundaries, raising the risk of corrosion penetration. Consequently, although Fe–Si alloying can improve some of the mechanical and processing properties of these composite materials, the introduction of these elements may simultaneously elevate their early-stage corrosion susceptibility by intensifying interfacial chloride adsorption.

Fe–Si doping systematically reduces the work function at all Cl^−^ adsorption sites on the Al(111)-Fe-Si/Al_2_Cu(110) interface compared to the corresponding undoped interface sites (Table 2), indicating effective modulation of the surface electrochemical activity. This reduction in the work function is attributed to the solid-solution incorporation or interfacial segregation of Fe and Si atoms, which alter the local hybridization environment of the adjacent Al and Cu atoms. The enhanced orbital overlap between these metal atoms and the adsorbed Cl^−^ strengthens their interaction and facilitates electron transfer from the substrate to the Cl^−^. Consequently, the overall surface electron-escape barrier declines and the interfacial dipole moment undergoes reconfiguration, leading to the observed decrease in the work function.

Although Fe–Si doping modifies the electronic characteristics of distinct adsorption sites on the Al(111)-Fe-Si/Al_2_Cu(110) interface, the decline in the work function is not uniform across all sites. Notably, at the Al-hole site, the work function decreases from 4.728 eV at the undoped Al(111)/Al_2_Cu(110) interface to 4.542 eV at the Fe–Si-doped interface. Coupled with the significantly enhanced adsorption energy at this location, this suggests that Fe–Si incorporation profoundly reconstructs the local atomic and electronic environment. This restructuring optimizes both the geometric coordination and charge distribution of the Al-hole site, leading to strengthened Cl^−^ adsorption while substantially lowering the electron binding energy of adjacent Al atoms. Consequently, the anodic dissolution tendency of the Al-hole site significantly increases, making this site a likely active center for pitting nucleation. By contrast, the Al-top site retains its intrinsically high reactivity, consistently displaying low values of the work function, and Fe–Si doping only leads to a very small decline in the work function. The persistently low work function of the Al-top site confirms its thermodynamic favorability for electron loss, indicating its role as an inherent corrosion-sensitive region on clean surfaces, interfaces, and doped interfaces alike.

At the Cu-hole site, the work function progressively decreases from 4.991 eV on the pristine Al_2_Cu(110) surface to 4.635 eV on the undoped Al(111)/Al_2_Cu(110) interface and to 4.591 eV on the Fe/Si-doped interface. This stepwise reduction indicates that the formation of the interface alone significantly activates the Cu-hole site, while Fe/Si alloying induces an additional, albeit subtle, electronic modulation. By contrast, the Cu-top site maintains a pronounced chemical inertness, exhibiting the highest work function across all configurations. Even in the doped interface, the work function of the Cu-top site is as high as 5.766 eV. This inertness emphasizes the fundamental role of the Cu-top site as a stable cathodic region largely unaffected by interface reconstruction or alloying. Thus, this site continues to function as an efficient cathode in localized corrosion cells. Moreover, the Cu-bridge site displays negligible changes in work function due to Fe/Si doping, reflecting its structural and electronic robustness within the interfacial environment.

This comprehensive analysis indicates that the intrinsic extremes of the adsorption sites on the interface are preserved by Fe/Si doping. Specifically, the high activity of the Al-top site and the high inertness of the Cu-top site are retained. Moreover, Fe/Si doping also restructures the interfacial electronic environment to expand the population of highly active anodic sites and intensifies their electrochemical activity without compromising the stability of the strong cathodic sites. The resulting microscopic spatial proximity of these highly active anodes and strong cathodes promotes the establishment of numerous efficient galvanic pairs across the interface. Consequently, the local electrochemical driving force is markedly amplified, resulting in intensified micro-galvanic coupling. This interfacial electronic reconstruction may lead to heightened susceptibility to early-stage localized corrosion at the macroscopic level.

**Figure 18:**
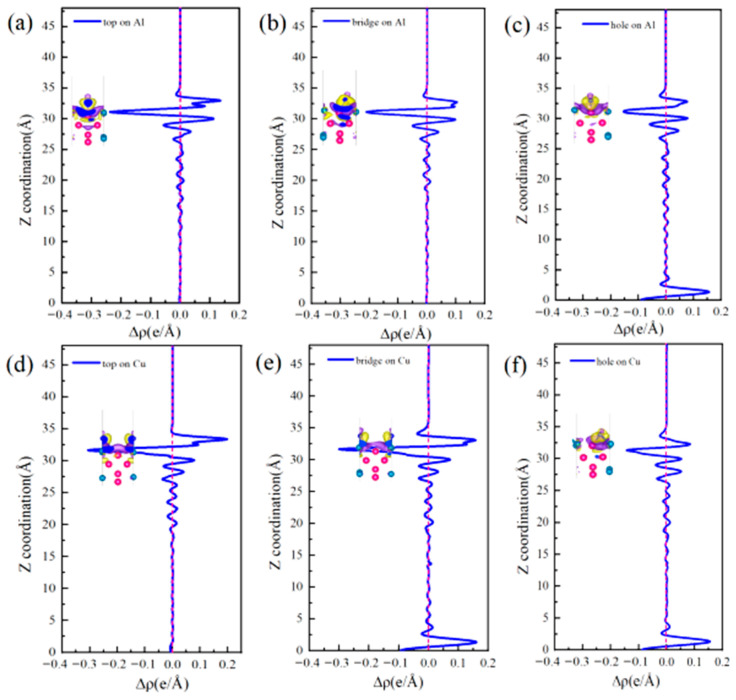
Differential charge densities of Cl^−^ adsorbed on the Al(111)-Fe-Si/Al_2_Cu(110) interface at different sites: (**a**) Al-top, (**b**) Al-bridge, (**c**) Al-hole, (**d**) Cu-top, (**e**) Cu-bridge, and (**f**) Cu-hole.

Figure 18 illustrates the variations in the differential charge density (Δρ) along the surface normal direction. The intensity of the negative Δρ peak, which reflects the axial gradient of electron density change, follows the order: top > bridge > hole. This trend is the opposite of that observed for global stability, as determined by the adsorption energy and work function (hole > bridge > top). Nevertheless, the two sequences are physically consistent and reflect complementary aspects of the adsorption-induced charge redistribution. The negative Δρ peak quantifies the degree of local charge depletion along a given axis, while the adsorption energy and work function describe the overall bonding strength and electrochemical activity of each site. Together, these observations affirm that multi-coordinated hole sites provide the most favorable adsorption centers at the interface, combining efficient spatial charge transfer with the highest thermodynamic stability.

At the top site, Cl^−^ interacts with a single substrate atom through a vertical “head-to-head” bond, leading to a highly localized charge distribution along the bond axis. This localization results in the sharpest electron-depletion peak observed in that specific direction, indicating strong local polarization and a correspondingly intense local electric field. However, this observation only represents a one-dimensional projection of the adsorption interaction. The true bond strength (adsorption energy) and the surface electrochemical activity (work function) were determined by the total charge transferred across the entire interaction volume and the integrated orbital-overlap efficiency.

Conversely, the hole sites exhibit a multi-dentate adsorption geometry that distributes charge transfer across three dimensions. Although the Δρ signal measured along any single axis appears smoothed or “diluted” due to this spatial delocalization, the cumulative electron transfer involving several neighboring atoms reaches a maximum. This multi-center cooperation enables more extensive orbital hybridization and electrostatic stabilization, eventually yielding the strongest adsorption bond (most negative adsorption energy) and the lowest electron-escape barrier (minimum work function) on a global scale. Essentially, the Δρ profile at the top site resembles a “deep, narrow probe,” highlighting the extreme local field strength, while that of the hole site resembles a “broad, shallow basin,” reflecting the spatially dispersed yet greater total charge redistribution. The latter determines the thermodynamic stability and electrochemical activity of each site.

The Δρ maps illustrate how efficient bonding at the hole sites is achieved through spatially delocalized charge transfer. Furthermore, the adsorption energy and work function values quantify the superior energetic stability and high reactivity of these sites. Together, these analyses establish an atomistic mechanism by which multi-coordinated hole sites serve as preferred locations for pitting nucleation at the interface.

**Figure 19:**
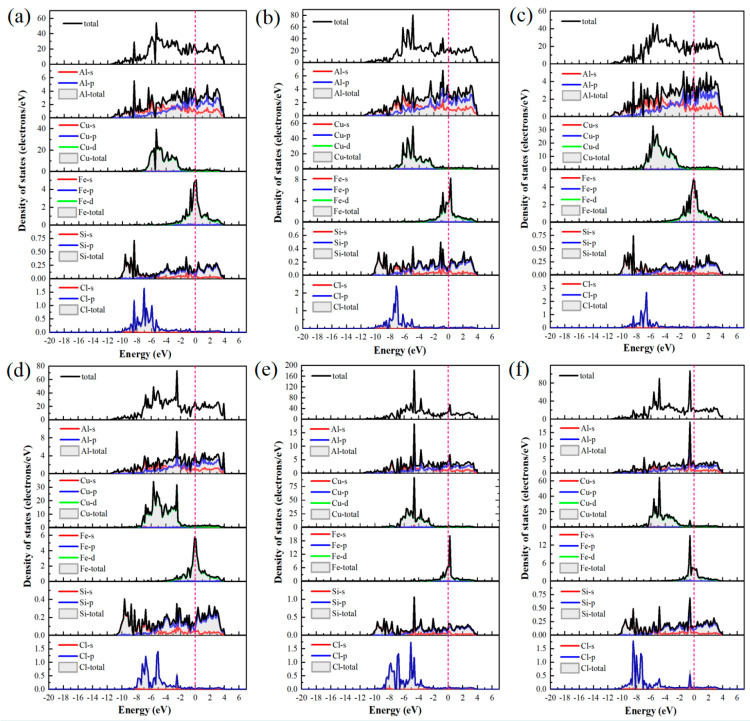
Density of states for Cl^−^ adsorbed on the Al(111)-Fe-Si/Al_2_Cu(110) interface at different sites: (**a**) Al-top, (**b**) Al-bridge, (**c**) Al-hole, (**d**) Cu-top, (**e**) Cu-bridge, and (**f**) Cu-hole.

Figure 19 depicts the PDOS for Cl^−^ adsorbed at various sites on the Al(111)-Fe-Si/Al_2_Cu(110) interface. Clearly, the Cl-3p orbitals provide a substantial contribution in the energy range of −9 to −4 eV below the E_F_, confirming their primary role in interfacial bonding. The Al-top and Al-bridge sites (Figure 19a,b) exhibit notably similar DOS. The Cl-3p orbitals for both sites exhibit a distinct bonding peak between −4 and −6 eV, which significantly overlaps with the Al-3p states. However, these two sites exhibit markedly lower peak intensity and sharpness than the Cu-top site. The total DOS near the E_F_ remains relatively high, indicating that Al–Cl hybridization and the associated charge transfer are weaker than those of Cu–Cl. This aligns well with the smaller negative differential charge density peaks observed for the Al-top and Al-bridge sites relative to the Cu-top site. Therefore, although charge is transferred from Al to Cl^−^ at the Al-top and Al-bridge sites, the interaction with Cl^−^ is less localized and intense than at the Cu-top site.

By contrast, the PDOS analysis (Figure 19c) shows that the Cl-3p peak at the Al-hole site is notably broad and extends from deep energies to near the E_F_, underscoring efficient multi-center coordination that enhances both orbital overlap and charge transfer. This electronic feature correlates with the highest adsorption stability and the lowest work function observed at this site. At the Cu-top site (Figure 19d), a sharp hybrid peak appears near −3 eV where the Cu-3d and Cl-3p contributions strongly overlap, indicating localized hybridization between these states. The concomitant suppression of the DOS near the E_F_ further confirms the stabilizing effect of this interaction. Together with the pronounced electron loss observed in the differential charge density, these results indicate the formation of a polarized, partially covalent bond involving significant local charge transfer from Cu to Cl^−^.

At the Cu-bridge site (Figure 19e), the Cl-3p contribution is distributed over a broad energy range from −7 to −5 eV, overlapping with Cu-3d, Al-3p, and Si-3p states. This behavior results in a diffuse hybrid peak in the PDOS. The intensity of PDOS remains relatively high close to the E_F_, suggesting the existence of accessible reactive states and a more delocalized, covalent-like bonding character. This feature is consistent with the partial back-donation of Cl^−^ lone-pair electrons into the metal bands.

At the Cu-hole site (Figure 19f), doping induces a downward shift in the Cl-3p peak and enhances its hybridization with Fe-3d, Al-3p, and Si-3p states. These orbital characteristics shift closer to the E_F_. The localized Fe-3d states facilitate charge exchange, and the Si-3p orbitals further regulate substrate reactivity via charge transfer. These modifications enhance charge-transfer efficiency and increase the PDOS intensity near the E_F_, thereby promoting Cl^−^ adsorption. The presence of a stabilized Cl-3p peak also suggests more favorable adsorption energetics.

These PDOS results collectively establish a unified electronic-structure framework that aligns with the trends observed for adsorption energy, work function, and differential charge density. This analysis clarifies why multi-coordinated sites, particularly the Al-hole site, exhibit the most stable bonding and enhanced electrochemical activity.

The impact of Fe and Si doping is evident in the PDOS. Fe-3d orbitals give rise to distinct localized features near the E_F_, associated with spatially confined electron states. When Cl^−^ is adsorbed at Cu sites (Figure 19d–f), the Fe-3d peaks are located close to or partially overlap with the Cu-3d band. This d–d orbital coupling widens the Cu d-band and shifts the d-band center to higher energies. Within the framework of the d-band center model, this upward shift can lead to a higher occupancy of metal–adsorbate antibonding states, potentially weakening the adsorption interaction. At strong adsorption sites such as the Cu-hole site, Fe doping attenuates and broadens the hybridization peak between Cl-p orbitals and metal d-bands. This indicates that Fe may partially passivate the Cu surface and reduce its intrinsic adsorption affinity for Cl^−^. Nonetheless, the localized Fe-3d states enable direct orbital hybridization with Cl-3p orbitals at the Cu-hole site.

For Cl^−^ adsorption at Al sites (Figure 19a–c), the PDOS exhibits distinct behavior. Al valence electrons reside predominantly in s and p orbitals, leading to a reduced PDOS intensity close to the E_F_. In the doped interface, adjacent Fe-3d states function as localized electron reservoirs that indirectly adjust the charge state of Al via charge transfer. These Fe-3d states may also provide additional orbitals for secondary orbital interaction with Cl electrons, thereby modulating Al–Cl bonding. The Si-3p orbital exhibits significant hybridization with both Al-3p and Cu-3d orbitals in the deep valence energy region. Owing to its higher electronegativity, Si draws electron density from neighboring Al and Fe atoms. This charge depletion reduces the electron density at adjacent metal sites, which in turn moderates their charge-transfer driving force toward electronegative Cl adsorbates during adsorption.

Briefly, Fe and Si dopants collectively influence the Cl^−^ adsorption behavior at the Al(111)-Fe-Si/Al_2_Cu(110) interface through local lattice distortion and charge redistribution, albeit via distinct electronic-structure modulation mechanisms. Fe primarily functions as an electronic structure modifier, with its localized 3d states significantly influencing the electronic structure. By coupling with the Cu-d band, Fe modifies the electronic properties of surface active centers and can directly participate in bonding. By contrast, Si acts as a charge-depleting element. High electronegativity of Si causes it to extract electrons from the surrounding matrix, which reduces the electron density of the substrate and its electron-donating propensity. Thus, charge transfer between the substrate and Cl^−^ is weakened, making the surface more inert.

These computational findings are consistent with the experimental observations reported in the literature. For instance, Li et al. [59] reported that Fe and Si impurities in the 7050 Al alloy formed insoluble cathodic phases, including Fe-rich intermetallics and Mg_2_Si, at grain boundaries. Notably, these phases act as microgalvanic cells that promote the initiation and acceleration of exfoliation corrosion. Increased concentrations of Fe and Si result in more severe corrosion damage and a transition in the corrosion morphology from mild pitting to extensive exfoliation with intergranular cracking, where corrosion severity scales with the areal fraction of these cathodic phases. Related experiments have also identified the Al_2_Cu/matrix interface as a preferential site for pit initiation in Cu–Al systems, with subsequent corrosion propagation along the interface followed by lateral invasion into the Al matrix [56]. These experimental findings confirm our computational analysis that Fe and Si dopants regulate interfacial electrochemical activity, and validate the crucial role of the Al/Al_2_Cu interface as a primary site for Cl^−^ accumulation and corrosion initiation. In this study, the comprehensive analysis of electronic structure thus provides a mechanistic interpretation for the impacts of Fe and Si doping and the distinct interfacial reactivity of the Al_2_Cu interface, establishing a coherent connection between atomic-scale calculations and experimentally observed corrosion behavior.

### 3.4. Influence of Stress on the Corrosion Performance of Al(111)-Fe-Si/Al_2_Cu(110)

Cu–Al laminated composites are often subjected to residual or applied mechanical stress in practical applications. This stress can lead to lattice distortion and altered atomic spacing, resulting in a significantly modified interfacial electronic structure [60,61]. Consequently, the adsorption of Cl^−^ and the subsequent initiation of corrosion processes are both influenced by the application of stress. In this study, systematic computational investigation on Cl^−^ adsorption across various sites on the Al(111)-Fe-Si/Al_2_Cu(110) interface reveals a clear distinction. That is, sites on the Al side generally exhibit strong Cl^−^ adsorption and low work functions, characteristics typical of active anodes, whereas the Cu-based sites tend to demonstrate greater chemical inertness. Two specific sites warrant further exploration regarding the effects of stress: the Al-bridge site, which displays a notable adsorption strength and a substantially lowered work function, and the Cu-hole site, whose electronic structure is significantly modulated by Fe/Si doping. Differential charge density and DOS analyses confirm that the Al-bridge site experiences pronounced charge transfer and orbital hybridization, identifying this site as a prototypical active anode crucial for the onset of corrosion. Moreover, both the adsorption energy and work function data of the Cu-hole site indicate its effective activation upon doping, while the DOS analysis reveals the direct involvement of Fe-3d and Si-3p orbitals. Thus, the Cu-hole site is an ideal candidate for exploring the coupling between alloying elements and the interfacial environment.

To probe the atomistic mechanisms underlying stress-mediated interfacial corrosion, chlorine adsorption was evaluated at the Al-bridge and Cu-hole sites under controlled mechanical strain. The Al(111)-Fe-Si/Al_2_Cu(110) interface was subjected to uniaxial strain along the X-direction, ranging from −5.0% (compression) to +5.0% (tension) in steps of 1.0%. The adsorption energy, work function, differential charge density, and DOS in the unstrained and strained states were then comparatively analyzed. This approach was employed to clarify how stress modulates interfacial atomic coordination, charge distribution, and orbital hybridization, and subsequently, how stress impacts chlorine adsorption stability, distribution of electrochemically active sites, and driving force for micro-galvanic corrosion. The results of this analysis provide an atomic-scale perspective for assessing the corrosion resistance of composite interfaces under realistic service conditions.

**Figure 20:**
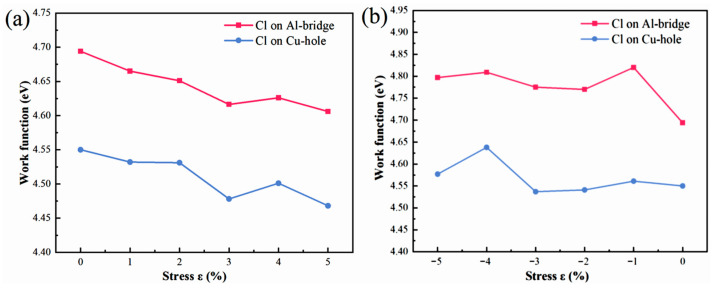
Effects of (**a**) tensile strain and (**b**) compressive strain on electron work function at different Cl^−^ adsorption sites on the Al(111)-Fe-Si/Al_2_Cu(110) interface.

When a material surface is subjected to mechanical strain, its atomic architecture alters, which can influence its corrosion properties. Moreover, the type, magnitude, and direction of stress produce distinct corrosion outcomes. The evolution of the electron work function for Cl^−^ adsorbed at the Al(111)-Fe-Si/Al_2_Cu(110) interface under tensile and compressive strains is presented in Figure 20. The figure illustrates that negative and positive signs denote compressive and tensile strain, respectively.

Under tensile strain, the work function of Cl^−^ adsorbed on the Al-bridge and Cu-hole sites experiences continuous decrease with increasing strain (ε) (Figure 20a). A lower work function indicates a reduced barrier for electron emission from the surface, implying a greater propensity for the surface to participate in anodic corrosion reactions. Therefore, a site with a lower work function exhibits higher corrosion activity. At both the Al-bridge and Cu-hole sites, the work function exhibits a brief, slight recovery under a strain of 4%, followed by a sharp drop at 5%. This non-monotonic variation closely parallels the experimental findings reported by Lü et al. [62], which state that “tensile stress disrupts passive films and promotes pitting corrosion”. The underlying mechanism is as follows: the interfacial atomic arrangement is disrupted by the tensile strain, which compromises the integrity of the passive layer. With the strain intensification, this passive film is ruptured more intensely, the surface electron-escape barrier is further lowered, and corrosion activity increases. However, corrosion products can accumulate over time to form a protective layer. For instance, Lü et al. reported that increasing the tensile stress of Cu–Al composite plates to approximately 2% of their yield strength (σ_s_) led to the formation of a dense corrosion-product film that effectively shielded the underlying metal from direct exposure to the corrosive medium. This reduced the corrosion rate and minimized mass loss, eventually manifesting as a decline in weight loss. In this study, the work function of Cl^−^ adsorbed at the Al-bridge site is consistently higher than that at the Cu-hole site. This indicates that the Al-bridge site possesses a stronger electron-escape capability and is more susceptible to corrosion, consistent with its role as a dominant anodic site during corrosion initiation.

Under compressive strain, the work function exhibits a non-monotonic, fluctuating decline with increasing strain (Figure 20b). This trend corresponds to the three-stage evolution mechanism of passive film formation under compression: densification, deformation-fracture, and finally plastic relaxation [62]. At low compressive stress, the densification of the passive film impedes the penetration and emission of electrons, resulting in an increase in the work function with increasing strain. This densified film effectively obstructs the penetration of Cl^−^ into the metal substrate, thereby maintaining the surface in a low-corrosion-activity state. When the compressive stress surpasses a critical threshold, excessive strain induces structural distortion and localized cracking of the passive film. This facilitates the penetration of Cl^−^ ions, significantly lowering the electron-escape barrier with the continuous increase in the strain. At this stage, the surface becomes markedly more active due to the deterioration of the corrosion-inhibiting function of the passive film. Once the compressive stress surpasses the yield strength, plastic deformation initiates stress relaxation, which alleviates further passive film cracking. Although the work function at the Al-bridge site remains relatively low, the overall integrity of the film is enhanced compared to the intermediate-pressure stage. Consequently, the corrosion rate decreases due to the inhibition of passive film cracking, and the work function initially increases slightly before gradually declining.

**Figure 21:**
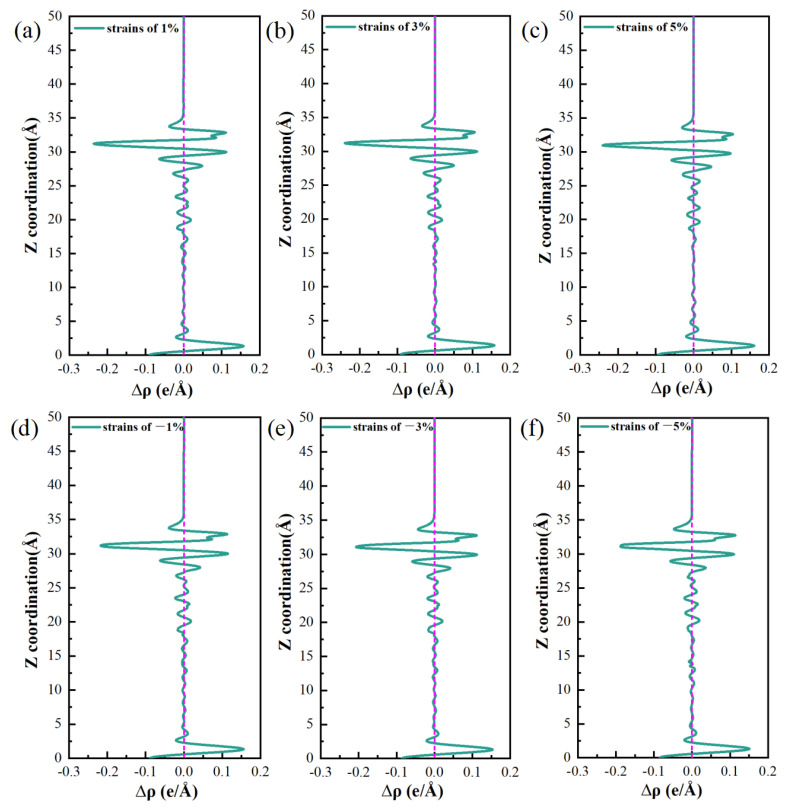
Effect of strain on differential charge density of Cl^−^ adsorbed at Al-bridge site on Al(111)-Fe-Si/Al_2_Cu(110) interface under strains of (**a**) +1%, (**b**) +3%, (**c**) +5%, (**d**) −1%, (**e**) −3%, and (**f**) −5%.

**Figure 22:**
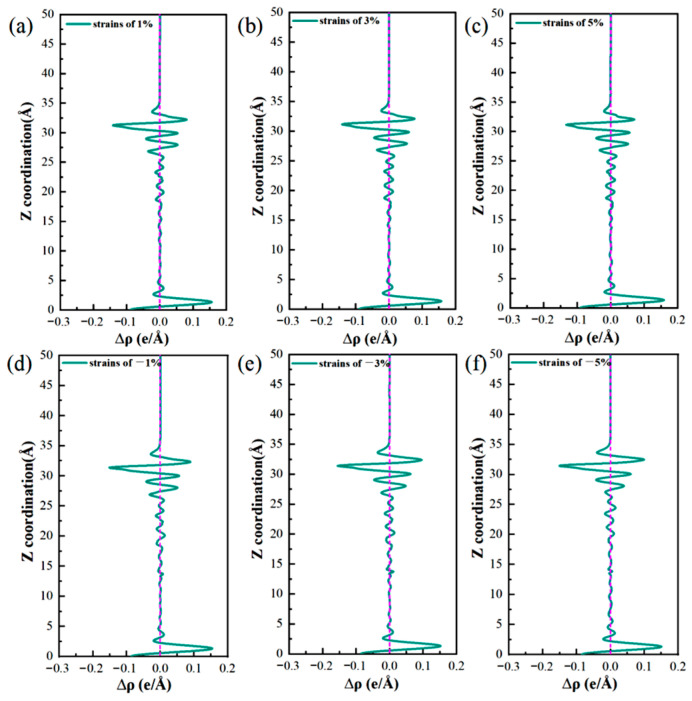
Effect of strain on differential charge density of Cl^−^ adsorbed at Cu-hole site on Al(111)-Fe-Si/Al_2_Cu(110) interface under strains of (**a**) +1%, (**b**) +3%, (**c**) +5%, (**d**) −1%, (**e**) −3%, and (**f**) −5%.

**Figure 23:**
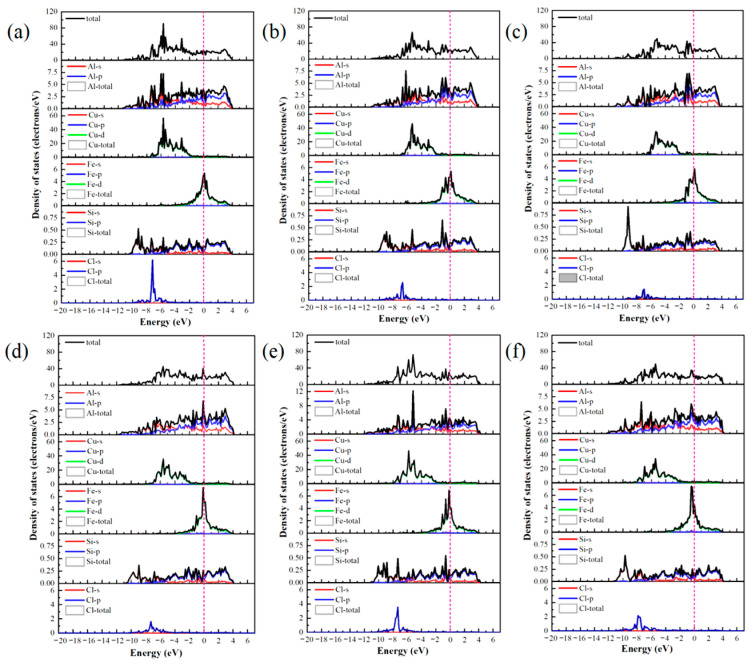
Effect of strain on the density of states of Cl^−^ adsorbed at Al-bridge site on the Al(111)-Fe-Si/Al_2_Cu(110) interface: (**a**) +1%, (**b**) +3%, (**c**) +5%, (**d**) −1%, (**e**) −3%, and (**f**) −5%.

**Figure 24:**
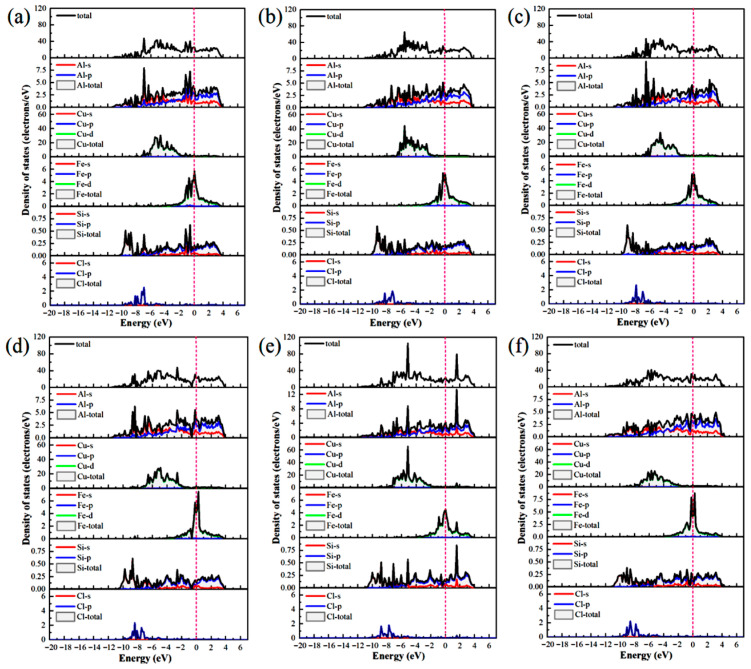
Effect of strain on the density of states of Cl^−^ adsorbed at Cu-hole site on the Al(111)-Fe-Si/Al_2_Cu(110) interface: (**a**) +1%, (**b**) +3%, (**c**) +5%, (**d**) −1%, (**e**) −3%, and (**f**) −5%.

The differential charge densities (Δρ) of Cl^−^ at the Al-bridge and Cu-hole sites on the Al(111)-Fe-Si/Al_2_Cu(110) interface as a function of strain (ranging from −5% to +5%) are illustrated in Figure 21 and Figure 22. The corresponding DOS evolution within the same strain range is depicted in Figure 23 and Figure 24. At the Al-bridge site, a pronounced electron accumulation peak (Δρ > 0) emerges near the adsorbed Cl^−^ under tensile strain. With the increase in the tensile strain from 1% to 5%, this peak becomes sharper, more localized, and more intense, while its width diminishes. This trend indicates that tensile elongation enhances the directional overlap between the Al-p and Cl-p orbitals, facilitating charge transfer from Al to Cl^−^. The resulting electron accumulation around Cl^−^ reinforces the ionic component of the Al–Cl bond and reduces the electron binding energy, consistent with the observed decrease in the work function. Furthermore, under compressive strain, an electron depletion valley manifests at the Cl^−^ adsorption site. With increasing compression from −1% to −5%, the depth of this valley initially rises before diminishing. Compression leads to an initial reduction in the Al–Cl bond distance, resulting in excessive orbital overlap. This favors electron back-donation from Cl^−^ to Al and creates a depletion region around Cl^−^. However, at higher compressive strains, atomic rearrangements promote charge delocalization, which mitigates this depletion effect.

At the Cu-hole site, only subtle variations in the differential charge density are observed under both tensile and compressive strain, indicating that the electronic distribution in this region is relatively insensitive to strain-induced perturbations. Throughout the loading process, the electron cloud surrounding the Cu-hole site remains remarkably stable, in contrast to the pronounced charge accumulation or depletion observed at the Al-bridge site. This stability can be attributed to the more complex multi-center bonding environment at Cu-hole sites, where competing atomic interactions effectively counteract strain-driven electronic configuration distortions.

Detailed DOS analysis was carried out to clarify the underlying strain-induced modulation mechanisms of interfacial bonding at various sites, based on orbital hybridization. At the Al-bridge site under tensile strain, the PDOS exhibits a reduced DOS near E_F_. This is accompanied by increased hybridization between Si-3p, Al-3p, and Fe-3d orbitals at higher energies, resulting in a new hybridization feature near E_F_. Concurrently, the contribution of Al-3d orbitals diminishes, resulting in a weaker hybridization with Cl-3p and causing a downward shift and reduction in the intensity of the Cl-3p peak. These observations suggest destabilization of the Al–Cl bond under tension. Moreover, although the Fe-3d contribution near the valence-band maximum increases, its overlap with Al-3d orbitals is reduced, suggesting that tensile strain also weakens Fe–Al orbital coupling. At the Cu-hole site, the PDOS exhibits a similar qualitative response to tensile strain but it is noticeably weaker, which aligns with the higher electronic stability of Cu-coordinated sites when subjected to mechanical deformation.

Under compressive strain, the PDOS near the E_F_ shows a non-monotonic variation, initially increasing and then decreasing with increasing strain. In this regime, Al-3p and Si-3p orbitals show enhanced combined hybridization at lower energies, with the corresponding hybrid peak becoming more intense under increased compression. This suggests that Si-3p orbitals contribute to modulating the covalent character of interfacial bonding. Concurrently, the Al-3p peak intensifies, reflecting enhanced hybridization with Cl-3p states, and the Cl-3p peak also shows an increase. While Fe-3d contributions remain limited at low energies, their hybridization with Al-3p near the E_F_ enhances moderately under compression, indicating that compressive strain promotes the coupling between transition-metal d-orbitals and modulates the local electronic structure. By contrast, the PDOS evolution at the Cu-hole site shows a more gradual change under compression, reflecting its lower sensitivity to strain compared to the Al-bridge site.

In summary, tensile strain weakens the bonding stability of the Al-bridge site by enhancing the orbital participation of Si and other dopants. Conversely, compressive strain modifies the interfacial electronic structure through increased covalent hybridization and d-orbital synergy. The Al-bridge site serves as the primary strain-responsive active center, whereas the Cu-hole site, despite being activated by doping, exhibits a relatively muted direct response to strain due to its unique electronic configuration.

This study employs an idealized sharp Al/Al_2_Cu interface model to characterize the fundamental electronic mechanisms governing how Fe/Si doping and strain modulate Cl^−^ adsorption. This simplification delineates the scope of the current study. Herein, coherent or incoherent interfacial structures, solute segregation, and composition gradients inherent to real materials are not included. The calculations are conducted in a vacuum without considering aqueous environments and competitive adsorbates such as OH^−^. Moreover, only the fully formed, stable Al_2_Cu phase is considered, and the Cu dissolution behavior is excluded. These factors collectively demonstrate the intricate coupled effects found in realistic corrosive environments. Building on this framework, future studies could advance to multiscale modeling. This could involve investigating the combined effects of interfacial misfit and segregation, integrating explicit solvation models to probe competitive adsorption, and developing composition-gradient models to evaluate the impact of dissolution limits. The atomic-scale mechanisms uncovered and the theoretical benchmark established in this study lay the groundwork for more practical engineering simulations.

## 4. Conclusions

This study elucidates the atomic-scale mechanisms governing corrosion initiation at the Al(111)/Al_2_Cu(110) interface through electronic structure analysis, revealing the synergistic roles of alloying elements (Fe, Si) and mechanical stress in modulating the interfacial corrosion behavior. The results provide theoretical guidance and novel strategies for enhancing the corrosion resistance of Cu–Al-laminated composites through rational composition design, interface engineering, and stress management. The main conclusions are as follows:

1. Upon Cl^−^ adsorption on the Al_2_Cu(110) surface, the Al sites exhibit strong localized charge transfer and a marked ionic character. Consequently, these sites serve as active centers for anodic dissolution. By contrast, Cu sites, in particular the Cu-top site, display delocalized covalent bonding and a high work function, functioning as inert cathodes. This “active anode and inert cathode” pairing directly establishes micro-galvanic couples that drive localized corrosion.
2. Owing to its more negative adsorption energy compared to that of Al_2_Cu(110), the Al(111)/Al_2_Cu(110) interface acts as a thermodynamic sink for Cl^−^ accumulation. At the nanoscale, high-activity anodic regions formed by multi-coordinated sites, such as the Al-hole site, coexist near the inert Cu-top cathode site. This configuration significantly enhances the micro-coupling effect, promoting preferential corrosion initiation and rapid propagation along the interface.
3. Silicon, which is characterized by its low substitution energy and high interfacial stability, effectively strengthens the interface. Conversely, iron primarily dissolves on the aluminum side. Although iron can modify the electronic structure, its introduction may lead to galvanic corrosion risks, and it does not reinforce the Al_2_Cu side. The synergistic effect of Fe/Si doping increases both the quantity and activity of highly reactive anodic sites, thereby amplifying the interfacial electrochemical contrast and the driving force for corrosion.
4. Tensile strain systematically enhances surface corrosion activity by lowering the work function, while compressive strain induces a non-monotonic variation in corrosion activity through a three-stage evolution of the passive film: “densification → rupture → plastic relaxation.”

## Figures and Tables

**Table 1:** Work functions of Cl^−^ adsorption on Al_2_Cu(110) surface and Al/Al_2_Cu interface.

Adsorption Site	Work Function (eV)	
	Al_2_Cu(110) Surface	Al/Al_2_Cu Interface
Al-top	4.993	4.974
Al-bridge	5.720	5.015
Al-hole	4.953	4.728
Cu-top	6.201	5.854
Cu-bridge	5.715	5.629
Cu-hole	4.991	4.635

**Table 2:** Work functions of Cl^−^ adsorption on Al(111)-Fe-Si/Al_2_Cu(110) interface.

Adsorption Site	Work Function (eV)		
	Al_2_Cu(110) Surface	Al/Al_2_Cu Interface	Al-Fe-Si/Al_2_Cu Interface
Al-top	4.993	4.974	4.963
Al-bridge	5.720	5.015	4.649
Al-hole	4.953	4.728	4.542
Cu-top	6.201	5.854	5.766
Cu-bridge	5.715	5.629	5.618
Cu-hole	4.991	4.635	4.591

## Data Availability

The original contributions presented in this study are included in the article. Further inquiries can be directed to the corresponding author.
